# Protocatechualdehyde ameliorates OGD/R-induced endothelial injury via the ROS/miR-29b-3p/SIRT1 regulatory axis

**DOI:** 10.3389/fphar.2026.1904530

**Published:** 2026-08-12

**Authors:** Yanqing Wu, Xilin Qiao, Shuaiyu Chen, Yalan Lu, Bingyan Mao, Mengdie Zhu, Xinzhi Chen, Nipi Chen, Zhishan Ding, Bo Jin

**Affiliations:** 1 School of Life Sciences, Zhejiang Chinese Medical University, Hangzhou, China; 2 School of Medical Technology and Information Engineering, Zhejiang Chinese Medical University, Hangzhou, China

**Keywords:** endothelial cells, miR-29b-3p, OGD/R, protocatechualdehyde, ROS

## Abstract

**Introduction:**

MicroRNA-29b-3p (miR-29b-3p) plays a critical role in regulating endothelial apoptosis and autophagy. We previously demonstrated that protocatechualdehyde (PCA), a water-soluble phenolic acid derived from *Salvia miltiorrhiza* Bge., protects human umbilical vein endothelial cells (HUVECs) against oxygen-glucose deprivation/reoxygenation (OGD/R)-induced injury. However, whether miR-29b-3p mediates this protective effect remains unclear.

**Methods:**

Using the OGD/R in vitro model of ischemia-reperfusion injury, we investigated the molecular mechanisms underlying ischemic damage at the cellular level. RT-qPCR was used to measure miR-29b-3p expression, Western blotting to assess autophagy- and apoptosis-related proteins, and MDC staining and flow cytometry to evaluate autophagy and apoptosis, respectively.

**Results:**

The results showed that miR-29b-3p was downregulated in OGD/R-treated HUVECs, an effect that was reversed by PCA and the ROS inhibitor N-acetylcysteine (NAC). The miR-29b-3p mimic enhanced the promoting effects of PCA and NAC on miR-29b-3p expression, whereas miR-29b-3p inhibitor reversed these effects. Similarly, PCA-induced autophagy and inhibition of apoptosis were enhanced by the miR-29b-3p mimic and reversed by the inhibitor. To determine whether SIRT1 acts downstream of miR-29b-3p, we used EX527 (a SIRT1 inhibitor) and found that it attenuated the PCA-mediated upregulation of miR-29b-3p. The miR-29b-3p mimic enhanced PCA-induced SIRT1 activity and protein expression, while the inhibitor had the opposite effect. Additional experiments revealed that NAC suppressed ROS by upregulating miR-29b-3p, leading to increased SIRT1 expression and reduced apoptosis.

**Disscussion:**

Collectively, these findings demonstrate that PCA ameliorates OGD/R-induced endothelial injury by promoting autophagy and inhibiting apoptosis via the ROS/miR-29b-3p/SIRT1 regulatory axis.

## Graphical Abstract

**Figure:**
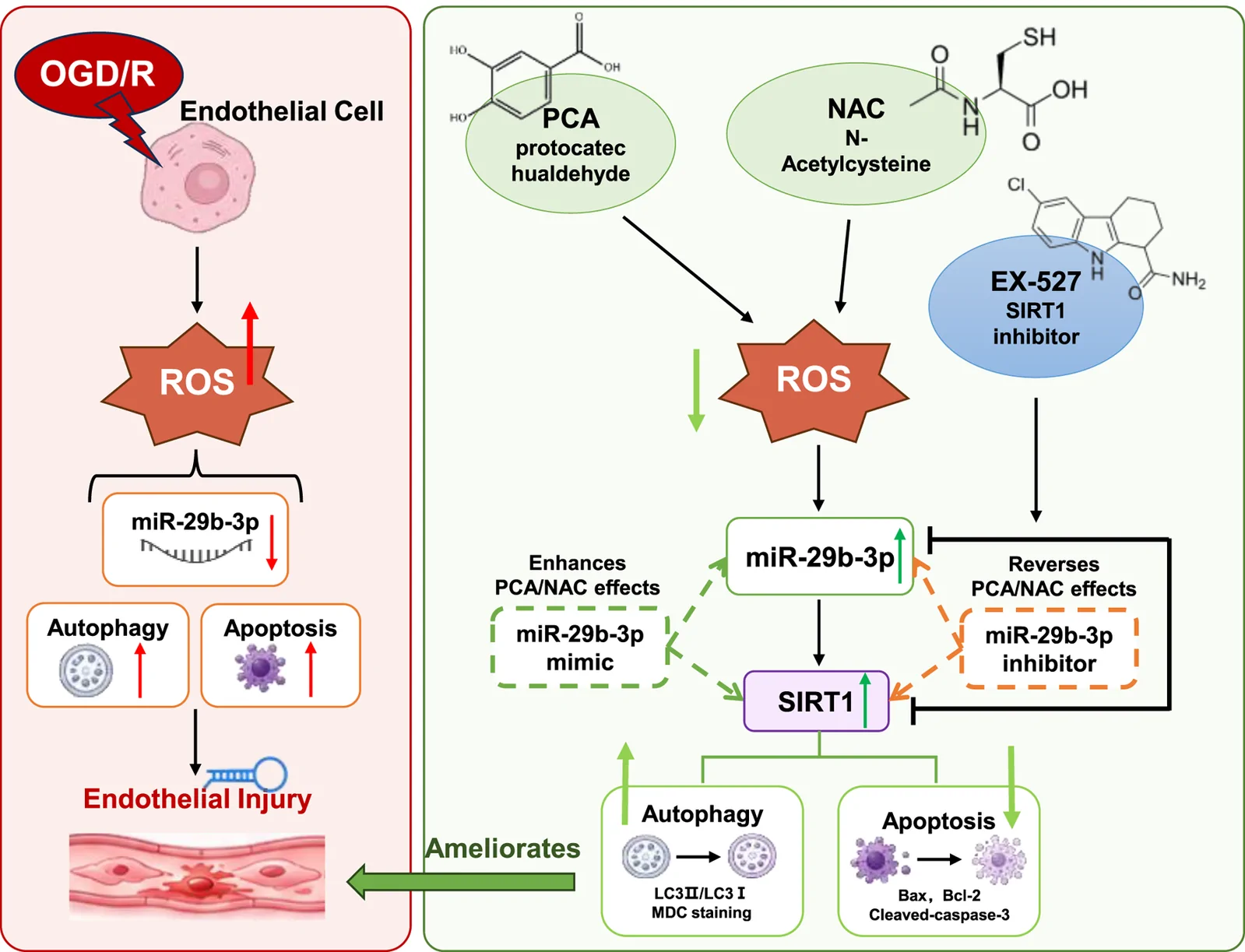

## 1 Introduction

Cardiovascular diseases remain the leading cause of morbidity, disability, and mortality worldwide, with acute myocardial infarction and ischemic cerebrovascular disease being the predominant contributors to mortality (Feng et al., 2019; Cao X. et al., 2022; Tapeinos et al., 2022). Although timely reperfusion is essential for salvaging ischemic tissue, it paradoxically induces ischemia-reperfusion (I/R) injury, which triggers endothelial dysfunction (Heusch and Gersh, 2017; Burda et al., 2023). This dysfunction manifests as apoptosis (Zhang et al., 2022a), oxidative stress (Xiang et al., 2021), impaired autophagy (Popov et al., 2023), and inflammation (Algoet et al., 2023). Endothelial cells (ECs) play a key role in maintaining vascular homeostasis. Dysfunctional ECs release pro-inflammatory factors that promote exacerbate damage to surrounding cells in the microcirculation (Zhang et al., 2022b). Therefore, preventing I/R-induced endothelial injury represents an effective therapeutic strategy for ischemic diseases.

Recently, the search for bioactive natural products with endothelial cytoprotective properties has gained increasing attention. Protocatechualdehyde (3,4-dihydroxybenzaldehyde; PCA) is a natural water-soluble polyphenol extracted from the roots of *Salvia miltiorrhiza* Bge. It has been shown to exert various pharmacological activities and to protect endothelial cells from I/R injury by inhibiting oxidative stress (Zhang et al., 2021), pyroptosis (Guo et al., 2022), and inflammation (Wang and Zhang, 2023). Our previous data demonstrated that PCA protected HUVECs from OGD/R-induced damage by promoting autophagy and inhibiting apoptosis via Sirtuin 1 (SIRT1) (Cao S. et al., 2022). Nevertheless, the upstream mechanism through which PCA upregulates SIRT1—particularly whether this effect is mediated by specific microRNAs—remains unknown.

Accumulating evidence indicates that natural compounds can protect ECs by regulating SIRT1 through microRNAs (miRNAs). SIRT1, an NAD^+^-dependent deacetylase, regulates transcriptional activity through histone deacetylation (O'Neill et al., 2026), and exerts antioxidant effects by deacetylating its targets (Imai, 2009). In addition, SIRT1 suppresses apoptosis by inhibiting p53 (Brunet et al., 2004). For example, cyanidin-3-O-glucoside attenuates TNF-α-induced apoptosis and inflammation in HUVECs by downregulating miR-204-5p and upregulating SIRT1 (Wang et al., 2022). Sulforaphane mitigates H_2_O_2_-induced endothelial damage through miR-34a repression and consequent SIRT1 upregulation, which reduces ROS production (Li et al., 2021). Similarly, ligustrazine ameliorated coronary microcirculation dysfunction by inhibiting miR-34a-5p, thereby enhancing SIRT1-mediated eNOS expression and suppressing NF-κB p65 acetylation (Gao et al., 2022). miR-29b-3p has been identified as regulator of endothelial cell function (Maucher et al., 2021) and is reported to suppress SIRT1 expression, and promote apoptosis (Zeng et al., 2020). However, whether this regulatory relationship applies to acute I/R injury, or whether PCA engages a distinct miR-29b-3p-dependent mechanism, remains to be determined.

In our preliminary screening using an RT-qPCR-based miRNA panel, miR-29b-3p emerged as the most markedly upregulated miRNA by PCA among the candidates examined. Given that miR-29b-3p has been implicated in the regulation of SIRT1 and endothelial apoptosis (Li et al., 2021), we hypothesized that PCA exerts its cytoprotective effects by upregulating miR-29b-3p, thereby reinforcing SIRT1-dependent anti-apoptotic and autophagic pathways. Therefore, this study aimed to investigate whether the miR-29b-3p/SIRT1 axis mediates the protective effects of PCA against OGD/R-induced injury in HUVECs.

## 2 Materials and methods

### 2.1 Cell culture and treatment

HUVECs were purchased from Chinese Academy of Sciences (Shanghai China) and cultured in RPMI 1640 Medium (Gibco, Waltham, United States) supplemented with 10% fetal bovine serum (Gemini, West Sacramento, United States), and 100 U/mL penicillin-streptomycin solution (Biosharp, Beijing, China) in a humidified incubator with 5% CO_2_ at 37 °C. Cells at passages 3–6 were used for all experiments. To minimize passage-related variability, cells at comparable passage numbers were used across all experimental groups, and key experiments were repeated with independently passaged cell batches.

To establish the OGD/R injury model, cells were incubated in sugar-free and serum-free MEM medium (Gibco, United States) and transferred to a hypoxia incubator chamber (Billups-Rothenberg, San Diego, United States) (94% N_2_, 5% CO_2_, 1% O_2_) at 37 °C for 10 h, followed by reoxygenation under standard culture condition for 24 h. To evaluate the effects of PCA, cells were treated with PCA (Aladdin Company, Shanghai, China; purity ≥99% by HPLC) in RPMI 1640 medium during the 24 h reoxygenation period.

For miRNA modulation, miR-29b-3p mimic, miR-29b-3p inhibitor and negative control random sequences were transferred into cells for 6 h prior to OGD/R treatment using Lipofectamine™ 3000 transfection reagent (Invitrogen, Waltham, United States) according to the manufacturer’s instructions. The final transfection concentrations were 50 nM for the miR-29b-3p mimic and 100 nM for the miR-29b-3p inhibitor. No significant differences were observed between the NC-transfected group and the non-transfected control.

### 2.2 Reverse transcription quantitative polymerase chain reaction (RT-qPCR)

Total RNA was extracted using the RNA-Quick Purification Kit (Yeasen Biotechnology, Shanghai, China). Reverse transcription was performed using the Mir-X™ miRNA First-Strand Synthesis Kit (Takara, Kyoto, Japan) using miRNA-specific stem-loop reverse transcription primers. Quantitative real-time PCR was then carried out on a StepOnePlus Real-Time PCR System using SYBR® Prime Ex Taq TM II (Tli RNase H Plus) (Takara, Kyoto, Japan) to quantify miR-29b-3p expression. The thermal cycling protocol was as follows: initial denaturation at 95 °C for 30 s, followed by 40 cycles of 95 °C for 5 s and 60 °C for 20 s. A dissociation curve analysis was included to verify amplification specificity. U6 snRNA served as the internal control for normalization (Fang et al., 2026; Wang et al., 2026), and relative expression was calculated using the 2^−ΔΔCt^ method. The forward primer sequences were as follows: miR-29b-3p: 5′-GCT​AGC​ACC​ATT​TGA​AAT​C-3′, U6: 5′-ATT​GGA​ACG​ATA​CAG​AGA​AGA​TT-3′. The reverse primers were provided with the Mir-X™ kit.

### 2.3 Cell viability assay

HUVECs were seeded in 96-well plates at 5,000/well. After treatment according to experimental design, cell viability was assessed using the CCK-8 assay kit (Yeasen Biotechnology, Shanghai, China) following the manufacturer’s protocol. Briefly, 10 μL CCK-8 solution was added to each well and incubated at 37 °C for 2 h. Absorbance was measured at 450 nm wavelength. All experiments were performed in triplicate with three technical replicates per condition.

### 2.4 SIRT1 activity assay

Cells were harvested and lysed after treatment, and SIRT1 activity was determined using a Human SIRT1 enzyme-linked immunosorbent assay (ELISA) kit (Lengton Bioscience, Shanghai, China), according to the manufacturer’s recommendations. Absorbances were measured at 450 nm. A seven-point standard curve (r^2^ > 0.99) was generated using recombinant standard proteins provided in the kit and served as a positive control for quantitative antigen-antibody binding.

### 2.5 Dual luciferase reporter assay

Dual-luciferase activity was measured using the Dual-Luciferase Reporter Assay System (Beyotime Biotechnology, Shanghai, China) following the manufacturer’s protocol. HEK-293T cells were co-transfected with the miR-29b-3p mimic or negative control, along with a firefly luciferase reporter plasmid carrying either the wild-type (WT) or mutant (MUT) 3′-UTR of SIRT1, and a Renilla luciferase plasmid for normalization. Luciferase activities were quantified 48 h post-transfection using a microplate reader.

### 2.6 Reactive oxygen species (ROS) and superoxide dismutase (SOD) activity detection

Intracellular ROS levels were measured using the fluorescent probe DCFH-DA (Beyotime Biotechnology, Shanghai, China). After treatment, cells were collected and incubated with DCFH-DA probe diluent at 37 °C for 20 min. Fluorescence images were captured using a fluorescent inverted microscope (Nikon, Kyoto, Japan), and fluorescence intensity was quantified using ImageJ software (excitation/emission: 488/525 nm). Rosup was used as a positive control to validate the ROS detection system.

Total SOD activity was determined using WST-8 method (Beyotime Biotechnology, Shanghai, China). Cells culture supernatants were collected according to the kit instructions, transferred to 96-well plates, and incubated at 37 °C for 30 min. Absorbance was measured at 450 nm.

### 2.7 Cell apoptosis assay

Apoptosis was assessed using the FITC Annexin-V Apoptosis Detection kit (BD Biosciences, Franklin Lakes, United States) according to the manufacturer’s instructions. Briefly, cells were harvested, and stained with 5 μL FITC-Annexin V and 5 μL PI at room temperature for 15 min in the dark. Apoptotic cells were analyzed using a flow cytometer (Beckman Coulter, Brea, United States). Staurosporine (1 μM) was used as a positive control.

### 2.8 Autophagy staining assay with monodansylcadaverine (MDC)

Autophagy was evaluated using a monodansylcadaverine (MDC) staining kit (Beyotime Biotechnology, Shanghai, China). Cells were collected and incubated with MDC staining solution at 37 °C for 30 min in the dark. Cells were then resuspended in assay buffer and analyzed by flow cytometry (excitation/emission: 335/518 nm). Rapamycin (100 nM) was used as a positive control.

### 2.9 Western blotting

Total protein was extracted using RIPA lysis buffer (Beyotime Biotechnology, Shanghai, China) supplemented with 1% PMSF and 2% phosphatase inhibitor cocktail. Protein concentrations were determined using the BCA protein assay Kit (Beyotime Biotechnology, Shanghai, China). Equal amounts of protein (30 μg per lane) were separated by 15% SDS-PAGE and transferred onto PVDF membranes (Millipore, Bedford, United States). Membranes were blocked with rapid blocking buffer (Beyotime Biotechnology, Shanghai, China) at room temperature for 20 min and then incubated with primary antibodies overnight at 4 °C. The following primary antibodies were used: anti-SIRT1 (1:2,000, cat# 13161-1-AP, Proteintech), anti-p62 (1:1,000, cat# 18420-1-AP, Proteintech, Chicago, United States), anti-Beclin1 (1:1,000, cat# 11306-1-AP, Proteintech), anti-LC3 (1:2,000, cat# 14600-1-AP, Proteintech, Chicago, United States), anti-Bcl2 (1:1,000, cat# 12789-1-AP, Proteintech), anti-Bax (1:1,000, cat# 50599-2-Ig, Proteintech, Chicago, United States), anti-β-actin (1:1,000, cat# 50599-2-Ig, Proteintech, Chicago, United States), anti-p53 (1:1,000, cat# YM3052, Immunoway, Suzhou, China), anti-Caspase-3 (1:1,000, cat# YM8058, Immunoway, Suzhou, China), and anti-cleaved Caspase-3 (1:1,000, cat# YC0006, Immunoway, Suzhou, China). After washing three times with 1× TBST (Tris-buffered saline with 0.1% Tween 20), membranes were incubated with HRP-conjugated goat anti-mouse IgG (1:10,000, cat# abs20163, Absin, Shanghai, China) or HRP-conjugated goat anti-rabbit IgG (1:10,000, cat# abs20147, Absin, Shanghai, China) for 2 h at room temperature. Protein bands were visualized using an ECL chemiluminescence kit (Beyotime Biotechnology, Shanghai, China) and detected with a ChemiScope 6000 chemiluminescence imaging system (Clinx, Shanghai, China). Band intensities were quantified using ImageJ software (version 1.5i, NIH, Bethesda, United States), with β-actin as the loading control.

### 2.10 Wound healing assay

HUVECs (5 × 10^5^ cells/well) were seeded in 6-well plates and cultured until they reached 80% confluence. A straight scratch was made using a 200 μL pipette tip, and detached cells were removed by washing twice with PBS. The medium was then replaced with serum-free medium containing the indicated treatments, and cells were cultured for additional 48 h. Images of the scratch area were captured at 0 and 48 h under an inverted microscope (Nikon, Japan) at ×100 magnification. Wound closure was quantified by measuring the scratch area using ImageJ software (version 1.5i), and the percentage of wound closure was calculated as [(area at 0 h − area at 48 h)/area at 0 h] × 100%.

### 2.11 Statistical analysis

All data are presented as mean ± standard deviation (SD) from at least three independent biological replicates. Representative images are from at least three independent experiments. Statistical analyses were performed using GraphPad Prism 10.1.2 software (GraphPad Software, San Diego, United States). For comparisons between two groups, an unpaired two-tailed Student’s t-test was used. For multiple group comparisons, one-way analysis of variance (ANOVA) followed by Tukey’s *post hoc* test was applied. *P* < 0.05 was considered statistically significant.

## 3 Results

### 3.1 PCA upregulated the expression level of miR-29b-3p in OGD/R-induced HUVECs

**FIGURE 1:**
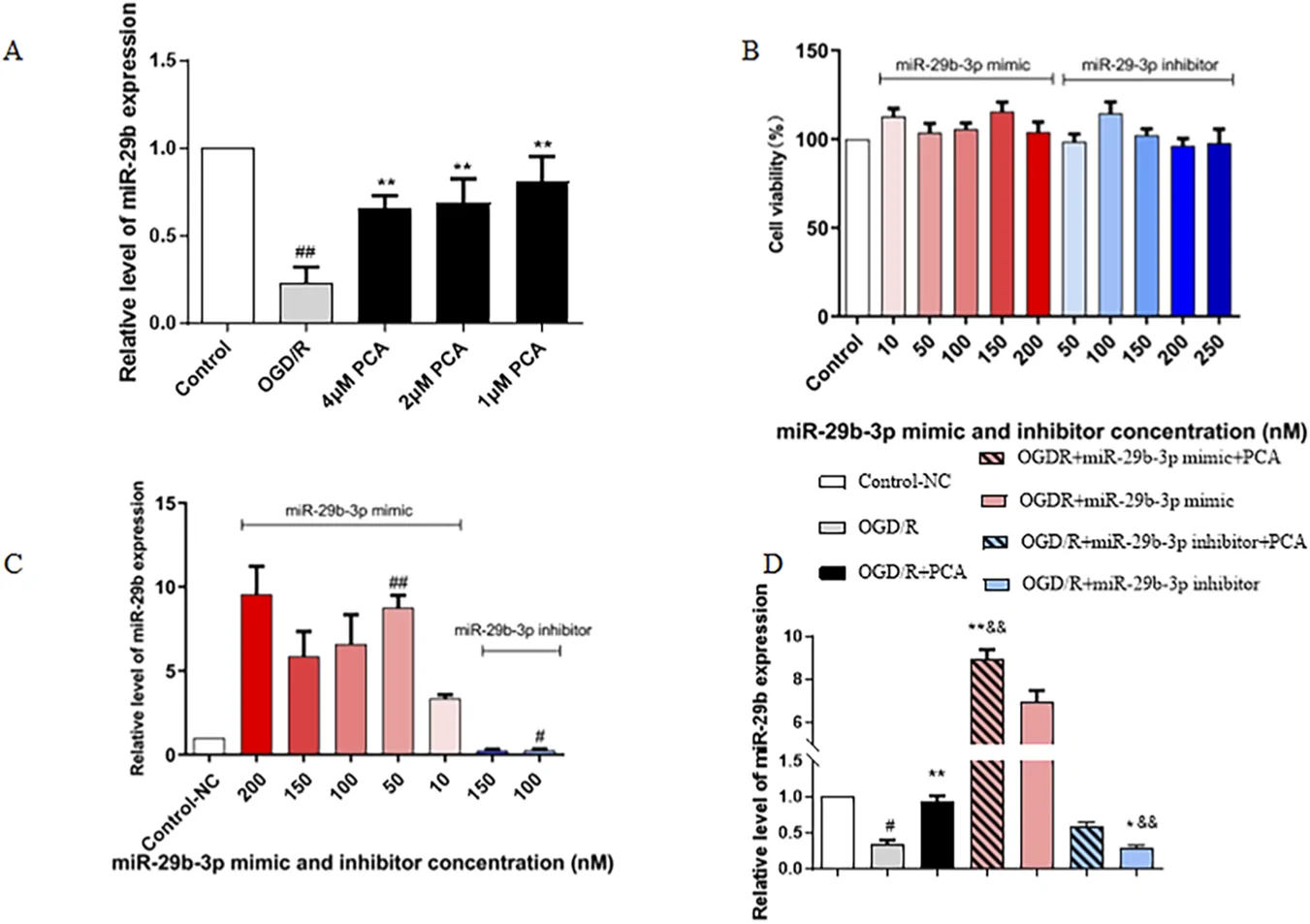
PCA upregulates miR-29b-3p expression in OGD/R-induced HUVECs. **(A)** HUVECs were subjected to OGD/R, and treated with PCA (1–4 μM) for 24 h miR-29b-3p expression was assessed by RT-qPCR. **(B)** HUVECs were transfected with increasing concentrations of miR-29b-3p mimic (10, 50, 100, 150, 200 nM) or miR-29b-3p inhibitor (50, 100, 150, 200, 250 nM) for 6 h Cell viability was evaluated by CCK-8 assay. **(C)** Transfection efficiency was validated by RT-qPCR. Based on these results, 50 nM mimic and 100 nM inhibitor were selected for subsequent experiments. **(D)** HUVECs were transfected with the miR-29b-3p mimic (50 nM) or inhibitor (100 nM) for 6 h, followed by OGD/R with or without PCA treatment. miR-29b-3p expression was measured by RT-qPCR. Data are presented as mean ± SD (n = 3 independent experiments). ^*#*^ *P <* 0.05, ^*##*^ *P <* 0.01 vs. control-NC group, ^*^ *P < 0.05*, ^**^ *P* < 0.01 vs. OGD/R group, ^*&&*^ *P* < 0.01 vs. PCA + OGD/R group.

We first evaluated the effect of PCA on miR-29b-3p expression in OGD/R-induced HUVECs using RT-qPCR (Figure 1A). Compared with the control group, miR-29b-3p expression was significantly reduced in HUVECs after OGD/R treatment. PCA (1, 2 and 4 μM) significantly upregulated miR-29b-3p expression in OGD/R induced HUVECs (*P <* 0.01). Although all three concentrations were effective, 1 μM was selected for subsequent mechanistic studies because it provided the optimal balance between miRNA modulation and cell viability; higher concentrations marginally reduced cell viability in the CCK-8 assay (Cao S. et al., 2022).

To further investigate the role of miR-29b-3p in PCA-mediated protection, HUVECs were transfected with miR-29b-3p mimic, inhibitor, or negative control (NC) prior to OGD/R exposure. The CCK-8 assay was employed to evaluate the cytotoxicity of the mimics and inhibitors in cells (Figure 1B), no significant differences were observed between the non-transfected OGD/R control and the NC-transfected group, and neither the mimic (10–200 nM) nor the inhibitor (50–250 nM) exhibited significant cytotoxicity. The optimal transfection concentration was then determined by RT-qPCR analysis (Figure 1C), 50 nM mimic and 100 nM inhibitor significantly modulated miR-29b-3p expression (*P <* 0.05) and were selected for subsequent experiments. As shown in Figure 1D, compared with 1 μM PCA alone, co-treatment with the miR-29b-3p mimic significantly enhanced PCA-induced miR-29b-3p upregulation in OGD/R-treated HUVECs (*P <* 0.01), whereas the miR-29b-3p inhibitor significantly reversed this effect (*P <* 0.01). Collectively, these results indicate that PCA significantly upregulates miR-29b-3p expression in OGD/R-injured HUVECs.

### 3.2 PCA promoted the proliferation and migration of HUVECs by upregulating miR-29b-3p

**FIGURE 2:**
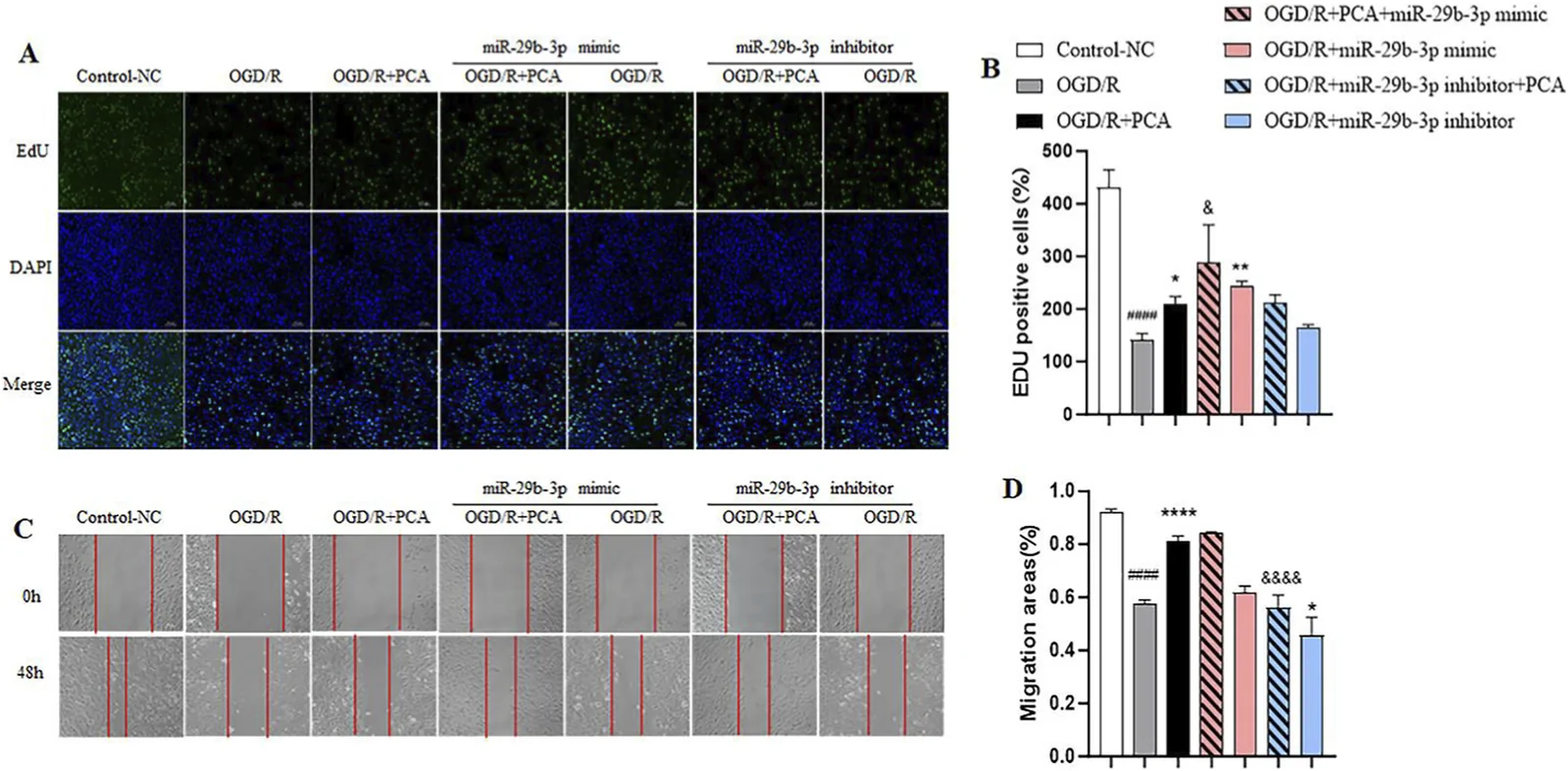
PCA promotes proliferation and migration of OGD/R induced HUVECs via miR-29b-3p. **(A)** Representative images of HUVEC proliferation (scale bar = 100 μm). **(B)** Quantification of cell proliferation using EdU assay. **(C)** Representative images of wound healing at 0 and 48 h (scale bar = 100 μm). **(D)** Quantification of wound closure percentage. HUVECs were transfected with miR-29b-3p mimic (50 nM) or inhibitor (100 nM) for 6 h, followed by OGD/R with or without PCA (1 μM) treatment. Data are presented as mean ± SD (n = 3 independent experiments). ^*####*^ *P < 0.0001* vs. control-NC group, ^*^ *P < 0.05,* ^**^ *P < 0.01,* ^****^ *P < 0.0001* vs. OGD/R group, ^*&*^ *P < 0.05,* ^&&&&^ *P < 0.0001* vs. 1 μΜ PCA + OGD/R group.

We next examined whether PCA promotes cell proliferation and migration via miR-29b-3p. As shown in Figure 2, compared with the OGD/R group, PCA significantly enhanced HUVECs proliferation (*P <* 0.05) and migration (*P* < 0.0001). Co-treatment with the miR-29b-3p mimic further increased both proliferation and migration compared with PCA alone (*P < 0.05*). In contrast, the miR-29b-3p inhibitor suppressed proliferation and migration, an effect that was partially reversed by co-treatment with PCA. These results demonstrated that PCA enhances HUVECs proliferation and migration by upregulating miR-29b-3p.

### 3.3 PCA enhanced antioxidant activity in OGD/R-induced HUVECs through miR-29b-3p

**FIGURE 3:**
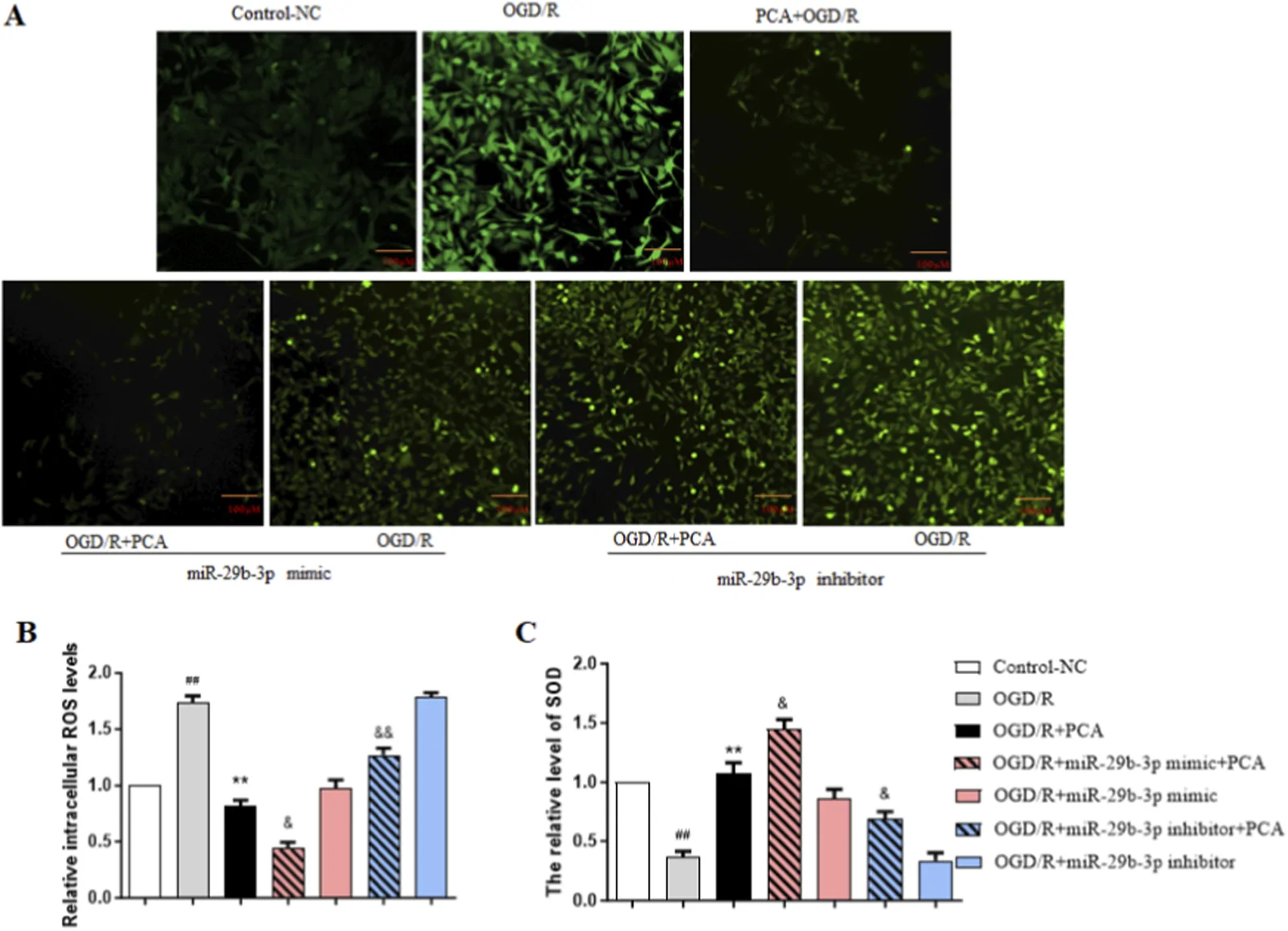
miR-29b-3p mediates the antioxidant effect of PCA in OGD/R-induced injury in HUVECs. HUVECs were transfected with miR-29b-3p mimic (50 nM) or miR-29b-3p inhibitor (100 nM) for 6 h, followed by OGD (10 h) and reoxygenation (24 h) with or without PCA (1 μM). **(A)** Representative fluorescent images of ROS stained with DCFH-DA (scale bar = 100 μm). **(B)** Quantification of intracellular ROS levels based on fluorescence intensity. **(C)** SOD activity measured using a total SOD assay kit. Data are presented as mean ± SD (n = 3 independent experiments). ^*##*^ *P <* 0.01 vs. control-NC group, ^**^ *P <* 0.01 vs. OGD/R group, ^*&*^ *P <* 0.05, ^*&&*^ *P <* 0.01 vs. 1 μM PCA + OGD/R group. Statistical analysis was performed using one-way ANOVA followed by Tukey’s *post hoc* test.

To investigate whether miR-29b-3p mediates the antioxidant effect of PCA in OGD/R-induced HUVECs, we measured intracellular ROS and SOD assays. As shown in Figures 3A,B, compared with the PCA + OGD/R group, co-treatment with the miR-29b-3p mimic significantly decreased the green fluorescence intensity (indicative of ROS levels), whereas the miR-29b-3p inhibitor significantly increased it (*P* < 0.05 and *P* < 0.01, respectively).

Furthermore, as shown in Figure 3C, the PCA-induced increase in SOD activity was enhanced by the miR-29b-3p mimic (*P* < 0.05) and abolished by miR-29b-3p inhibitor (*P* < 0.05). Collectively, these findings demonstrate that miR-29b-3p modulates the antioxidant effect of PCA in OGD/R-injured HUVECs.

### 3.4 PCA inhibited apoptosis in OGD/R-induced HUVECs through miR-29b-3p

**FIGURE 4:**
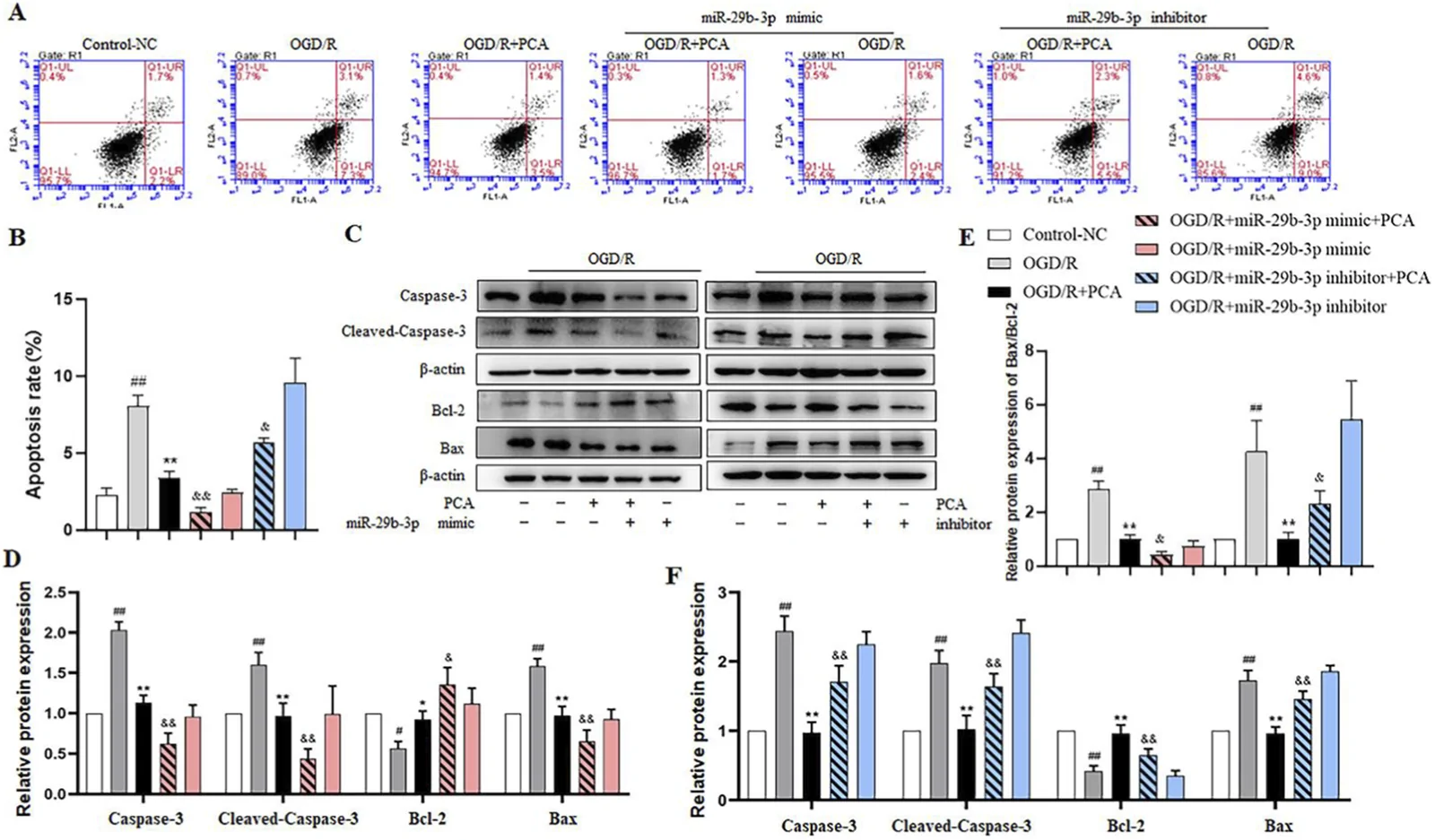
miR-29b-3p mediates the anti-apoptotic effect of PCA in OGD/R-induced HUVECs. HUVECs were transfected with miR-29b-3p mimic (50 nM) or inhibitor (100 nM) for 6 h, followed by OGD (10 h) and reoxygenation (24 h) with or without PCA (1 μM). **(A)** Representative flow cytometry plots of Annexin V-FITC/PI staining. **(B)** Quantification of apoptotic cells (early and late apoptosis combined). **(C)** Representative Western blots of apoptosis-related proteins. **(D–F)** Quantification of Bax, Bcl-2, Bax/Bcl-2 ratio, Caspase-3, and cleaved Caspase-3 levels, normalized to β-actin. Data are presented as mean ± SD (n = 3 independent experiments). ^*#*^ *P <* 0.05, ^*##*^ *P <* 0.01 vs. control-NC, ^*^ *P <* 0.05, ^**^ *P <* 0.01 vs. OGD/R, ^*&*^ *P <* 0.05, ^*&&*^ *P <* 0.01 vs. 1 μM PCA + OGD/R.

We next investigated whether miR-29b-3p regulates the anti-apoptotic effect of PCA in OGD/R-induced HUVECs using flow cytometry. As shown in Figures 4A,B, PCA treatment alone reduced the apoptosis rate to 3.5% ± 0.1% compared with the OGD/R group. Co-treatment with the miR-29b-3p mimic further decreased the apoptosis rate to 1.7% ± 0.2% (*P* < 0.01 vs. PCA alone), whereas the miR-29b-3p inhibitor reversed the anti-apoptotic effect of PCA, increased the apoptosis rate to 5.5% ± 0.4% (*P* < 0.01 vs. PCA alone).

Western blotting analysis yielded consistent results (Figures 4C–F). Compared with the PCA + OGD/R group, co-treatment with the miR-29b-3p mimic significantly enhanced the inhibitory effect of PCA on apoptosis: the expression levels of the pro-apoptotic proteins Caspase-3, cleaved-Caspase3, and Bax, as well as the Bax/Bcl-2 ratio were reduced, while the expression of the anti-apoptotic protein Bcl-2 was significantly increased. In contrast, the miR-29b-3p inhibitor significantly attenuated these effects of PCA. Collectively, these results demonstrate that PCA alleviates OGD/R-induced apoptosis by upregulating miR-29b-3p.

### 3.5 PCA promoted autophagy in OGD/R-induced HUVECs through miR-29b-3p

**FIGURE 5:**
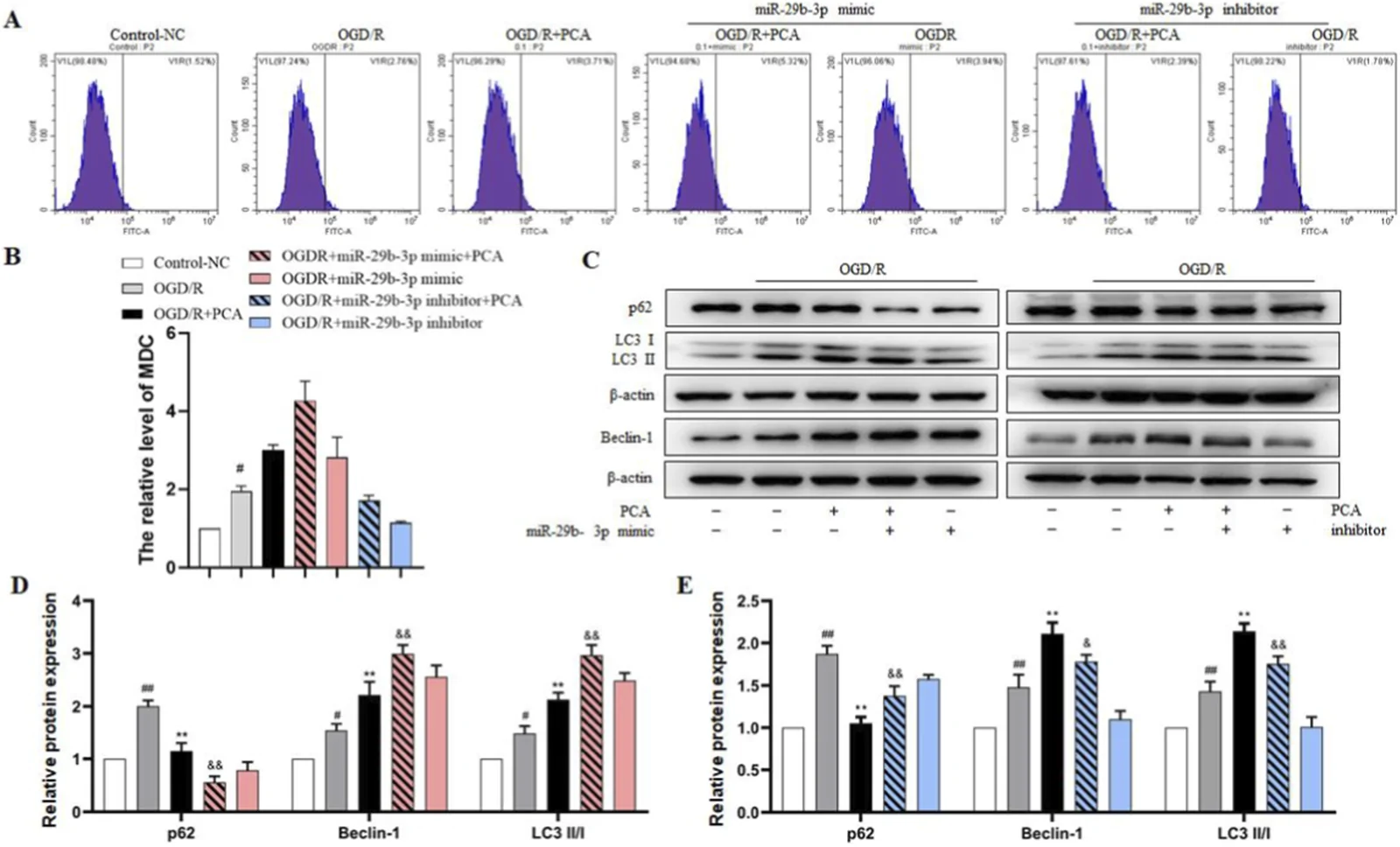
miR-29b-3p mediates the pro-autophagic effect of PCA in OGD/R-induced injury in HUVECs. HUVECs were transfected with miR-29b-3p mimic (50 nM) or inhibitor (100 nM) for 6 h, followed by OGD (10 h) and reoxygenation (24 h) with or without PCA (1 μM). **(A)** Representative flow cytometry histograms of MDC staining. **(B)** Quantification of MDC-positive cells (autophagy rate). **(C)** Representative Western blots of autophagy-related proteins. **(D,E)** Quantification of p62, Beclin1, and LC3-II/LC3-I ratio levels, normalized to β-actin. Data are presented as mean ± SD (n = 3 independent experiments). ^*#*^ *P <* 0.05, ^*##*^ *P <* 0.01 vs. control-NC, ^*^ *P < 0.05*, ^**^ *P < 0.01* vs. OGD/R, ^*&&*^ *P <* 0.01 vs. 1 μM PCA + OGD/R.

To determine whether miR-29b-3p mediates the pro-autophagic effect of PCA in OGD/R-induced HUVECs, we performed an MDC staining assay. As shown in Figures 5A,B, compared with the PCA + OGD/R group (3.71% ± 0.2%), co-treatment with the miR-29b-3p mimic increased the autophagy rate to 5.32% ± 0.1% (*P* < 0.01), whereas the miR-29b-3p inhibitor decreased it to 2.39% ± 0.4% (*P* < 0.01).

We further examined the expression of autophagy-related proteins by Western blotting. As shown in Figure 5C, the miR-29b-3p mimic markedly enhanced the PCA-induced changes in Beclin1, p62, and the LC3-II/LC3-I ratio (quantified in Figure 5D). In contrast, the miR-29b-3p inhibitor reversed these effects of PCA (Figure 5E). Taken together, these results indicate that PCA promotes autophagy in OGD/R-injured HUVECs via miR-29b-3p.

### 3.6 miR-29b-3p expression can affect the regulation of SIRT1 expression by PCA

**FIGURE 6:**
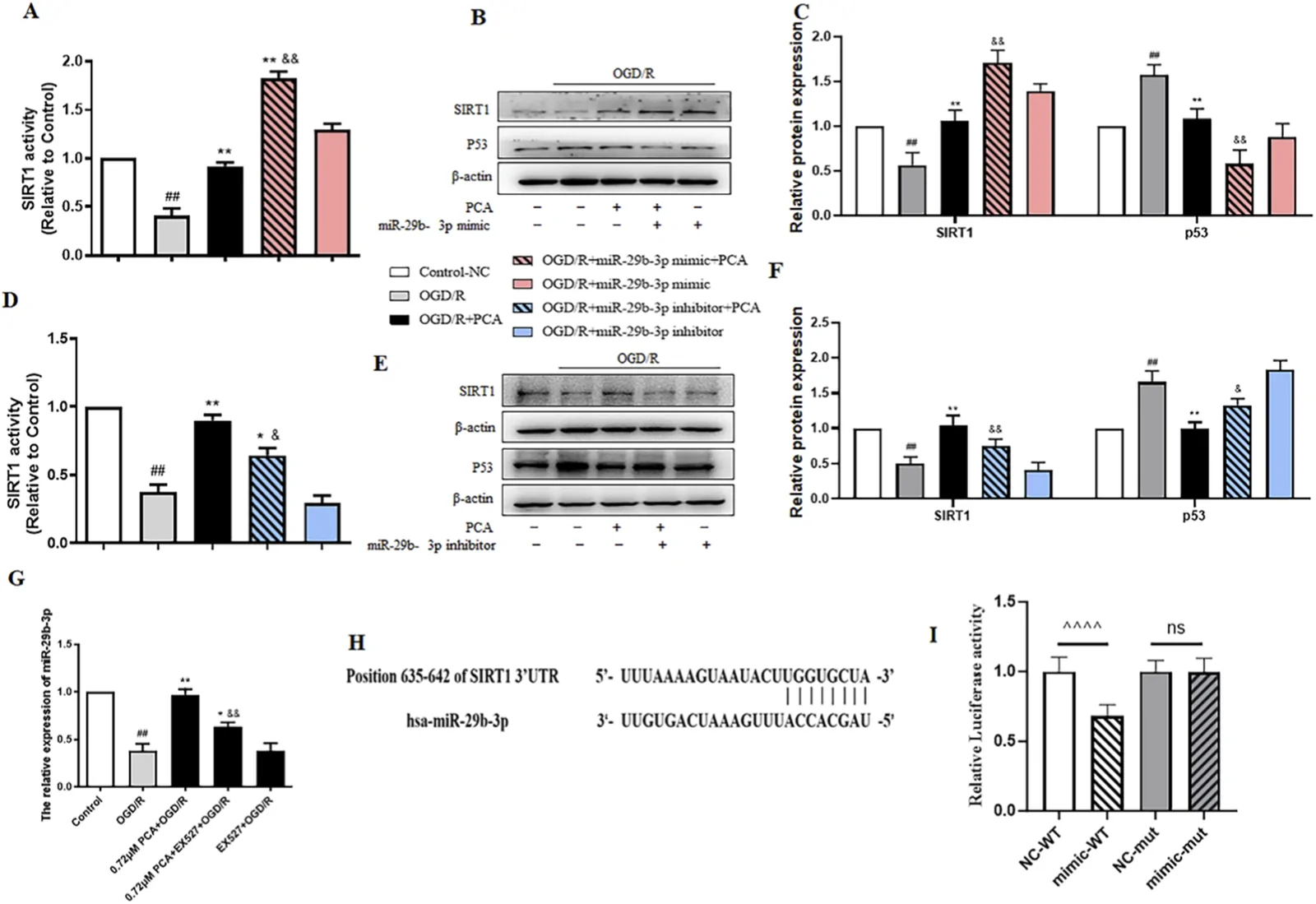
miR-29b-3p regulates SIRT1 through direct 3-UTR binding and reciprocal regulation in OGD/R-injured HUVECs. HUVECs were transfected with miR-29b-3p mimic (50 nM) or inhibitor (100 nM) for 6 h, followed by OGD/R with or without PCA (1 μM) treatment. SIRT1 activity was measured by ELISA, and SIRT1 protein expression was detected by Western blotting. **(A)** Quantification of SIRT1 activity in cells treated with miR-29b-3p mimic. **(B)** Representative Western blots of SIRT1 and p53 expression in cells treated with miR-29b-3p mimic. **(C)** Quantification of protein expression (normalized to β-actin) from **(B)**. **(D)** Quantification of SIRT1 activity in cells treated with miR-29b-3p inhibitor. **(E)** Representative Western blots of SIRT1 and p53 expression in cells treated with miR-29b-3p inhibitor. **(F)** Quantification of protein expression (normalized to β-actin) from **(E)**. **(G)** HUVECs were treated with EX527 (a SIRT1 inhibitor, 1 μM) for 24 h, with or without PCA (1 μM), and miR-29b-3p expression was measured by RT-qPCR. **(H)** Schematic diagram of the predicted miR-29b-3p binding site within the wild-type (WT) 3′-UTR of SIRT1 and the corresponding mutant (MUT) sequence. **(I)** Dual-luciferase reporter assay in HEK-293T cells co-transfected with miR-29b-3p mimic or NC, together with firefly luciferase reporters carrying either the WT or MUT 3′-UTR of SIRT1, and a Renilla luciferase plasmid for normalization. Luciferase activities were measured 48 h post-transfection. Data are presented as mean ± SD (n = 3 independent experiments). ^*##*^ *P <* 0.01 vs. control-NC, ^*^ *P <* 0.05, ^**^ *P <* 0.01 vs. OGD/R, ^*&*^ *P <* 0.05, ^*&&*^ *P <* 0.01 vs. 1 μM PCA + OGD/R, ^*^^^^*^ *P <* 0.0001 vs. NC-WT.

To determine whether miR-29b-3p regulates SIRT1 expression, HUVECs were transfected with miR-29b-3p mimic or inhibitor. As shown in Figure 6A, compared with PCA treatment alone, the miR-29b-3p mimic significantly enhanced the PCA-induced increase in SIRT1 activity (*P <* 0.01). Consistently, Western blotting revealed that the miR-29b-3p mimic further enhanced the PCA-induced upregulation of SIRT1 protein expression and inhibitory effect on p53, a well-known SIRT1 substrate (Figures 6B,C, *P <* 0.01). In contrast, the miR-29b-3p inhibitor markedly reversed these effects of PCA, as demonstrated by decreased SIRT1 activity (Figure 6D, *P <* 0.05) and increased p53 expression (*P < 0.05*) accompanied by reduced SIRT1 protein levels (Figures 6E,F, *P <* 0.01). These results indicate that miR-29b-3p positively regulates SIRT1 expression in OGD/R-injured HUVECs.

We next investigated whether SIRT1 conversely regulates miR-29b-3p. As shown in Figure 6G, treatment with EX527, a specific SIRT1 inhibitor, significantly reduced miR-29b-3p expression and attenuated the PCA-induced upregulation of miR-29b-3p (*P <* 0.01), suggesting that SIRT1 acts upstream of miR-29b-3p.

To further confirm whether miR-29b-3p directly targets SIRT1, dual-luciferase reporter assays were performed in HEK-293T cells. After 48 h of co-transfection, miR-29b-3p mimic significantly reduced the luciferase activity of the wild-type (WT) reporter, but exerted no inhibitory effect on the mutant (MUT) reporter (Figures 6H,I), confirming a potential direct interaction between miR-29b-3p and SIRT1 3′-UTR. Collectively, these results reveal a reciprocal regulatory relationship between miR-29b-3p and SIRT1, through which PCA reinforces SIRT1-mediated cytoprotective effects in OGD/R-injured HUVECs.

### 3.7 Inhibition of ROS can upregulate miR-29b-3p, inhibit apoptosis, and promote the expression of SIRT1

Our results above demonstrated that PCA significantly reduced intracellular ROS levels after OGD/R injury. Given the pleiotropic effects of ROS, we further used the ROS inhibitor N-acetylcysteine (NAC) to validate the involvement of ROS in the regulation of the miR-29b-3p/SIRT1 axis. The optimal concentration of NAC (1 mM) was determined by CCK-8 assay (data not shown).

**FIGURE 7:**
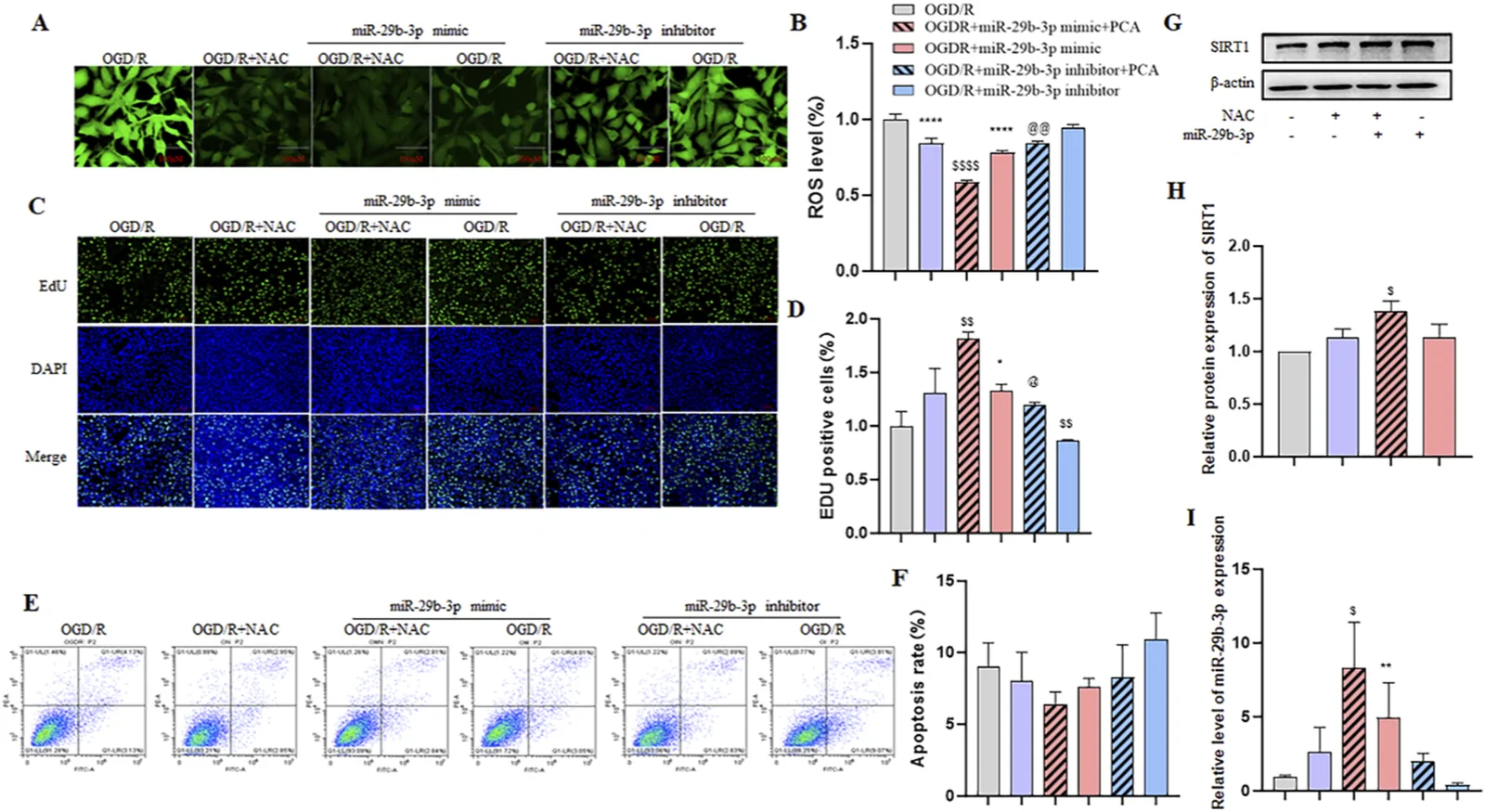
The ROS inhibitor NAC upregulates miR-29b-3p expression, suppresses ROS production and apoptosis, and promotes proliferation and SIRT1 expression in OGD/R-injured HUVECs. HUVECs were transfected with miR-29b-3p mimic (50 nM) or inhibitor (100 nM) for 6 h, followed by OGD/R with or without NAC (1 mM) treatment. **(A)** Representative fluorescent images of ROS stained with DCFH-DA (scale bar = 100 μm). **(B)** Quantification of intracellular ROS levels. **(C)** Representative images of cell proliferation (scale bar = 100 μm). **(D)** Quantification of cell proliferation by CCK-8 assay. **(E)** Representative flow cytometry plots of Annexin V-FITC/PI staining. **(F)** Quantification of apoptotic cells. **(G)** Representative Western blots of SIRT1. **(H)** Quantification of SIRT1 expression, normalized to β-actin. **(I)** miR-29b-3p expression measured by RT-qPCR. Data are presented as mean ± SD (n = 3 independent experiments). ^*^ *P <* 0.05, ^**^ *P <* 0.01*,* ^****^ *P <* 0.0001 vs. OGD/R, ^*$*^ *P <* 0.05, ^*$$*^ *P <* 0.01*,* ^$$$$^ *P <* 0.0001 vs. OGD/R + miR-29b-3p mimic, ^*@*^ *P <* 0.05, ^*@@*^ *P <* 0.01, vs. OGD/R + miR-29b-3p inhibitor. Statistical analysis was performed using one-way ANOVA followed by Tukey’s *post hoc* test.

As shown in Figures 7A,B, NAC treatment alone significantly reduced OGD/R-induced ROS levels (*P <* 0.0001). Co-treatment with NAC and the miR-29b-3p mimic further decreased ROS levels compared with the mimic alone (*P <* 0.0001). Conversely, the miR-29b-3p inhibitor increased ROS levels, an effect that was significantly reversed by co-treatment with NAC (*P <* 0.01).

NAC treatment also significantly increased HUVEC proliferation compared with the OGD/R group (Figures 7C,D). Co-treatment with NAC and the miR-29b-3p mimic further enhanced proliferation compared with the mimic alone (*P <* 0.01). Moreover, NAC reversed the decrease in proliferation caused by the miR-29b-3p inhibitor (*P <* 0.05).

The flow cytometry results (Figures 7E,F) showed that NAC treatment reduced the apoptosis rate from 7.26% ± 0.4%–5.8% ± 0.3% compared with the OGD/R group. Co-treatment with NAC and the miR-29b-3p mimic further decreased apoptosis compared with the mimic alone, whereas NAC reversed the pro-apoptotic effect of the miR-29b-3p inhibitor, reducing the apoptosis rate from 12.98% ± 0.4%–5.72% ± 0.3%.

Western blotting analysis (Figures 7G,H) revealed that both NAC and the miR-29b-3p mimic increased SIRT1 expression compared with the OGD/R group. Notably, NAC further enhanced the SIRT1 upregulation induced by the miR-29b-3p mimic (*P* < 0.05).

RT-qPCR results (Figure 7I) demonstrated that NAC significantly enhanced the miR-29b-3p upregulation induced by the miR-29b-3p mimic (*P* < 0.05) and reversed the inhibitory effect of the miR-29b-3p inhibitor. Collectively, these results indicate that inhibition of OGD/R-induced ROS by NAC upregulates miR-29b-3p expression, promotes SIRT1 expression, and inhibits apoptosis in OGD/R-injured HUVECs.

## 4 Discussion

The present study was designed to elucidate whether PCA enhances autophagy and inhibits apoptosis by upregulating miR-29b-3p, thereby ameliorating OGD/R-induced endothelial injury. miRNAs regulate diverse biological processes, and accumulating evidence indicates that the protective effects of natural compounds on endothelial cells are often mediated through miRNA modulation. Previous studies have established that miR-29b-3p is involved in multiple regulatory pathways in cardiovascular diseases, and its downregulation has been implicated in the pathogenesis of I/R injury (He and Yan, 2021). For instance, Han et al. reported that low expression of miR-29b-3p contributed to the deterioration of myocardial I/R injury, exacerbating pathological changes, fibrosis, apoptosis, and oxidative stress in both *in vitro* and *in vivo* models (Han et al., 2022). Consistent with these findings, we observed that OGD/R treatment significantly downregulated miR-29b-3p expression in HUVECs, supporting a protective role for miR-29b-3p in I/R injury.

Our results further demonstrated that PCA treatment markedly increased miR-29b-3p expression, and that overexpression of miR-29b-3p potentiated this effect. Previous studies have shown that inhibition of miR-29b-3p expression significantly increases oxidative stress and promotes apoptosis in endothelial cells, whereas upregulation of miR-29b-3p suppresses the progression of atherosclerosis, alleviates oxidative stress injury, and improves endothelial function (Zhou et al., 2023; Tong et al., 2022). Consistently, our data showed that, on top of PCA treatment, miR-29b-3p overexpression reduced ROS levels and enhanced SOD activity, while miR-29b-3p inhibition produced opposite effects. Similar results were obtained for apoptosis and autophagy. Collectively, these findings demonstrate, for the first time, that the protective effect of PCA against OGD/R-induced HUVEC injury is mediated by miR-29b-3p.

Reactive oxygen species (ROS) are major mediators of oxidative stress. Our results indicated that PCA significantly inhibited OGD/R-induced excessive ROS production by upregulating miR-29b-3p, thereby alleviating oxidative damage. Supporting this notion, treatment with the ROS inhibitor NAC not only suppressed ROS but also upregulated miR-29b-3p. Moreover, combined treatment with NAC and the miR-29b-3p mimic recapitulated the effects of PCA, reducing oxidative stress and inhibiting apoptosis. These findings suggest that ROS may act upstream of miR-29b-3p in mediating the protective effects of PCA.

SIRT1 has been identified as a direct target of miR-29b-3p in various biological contexts, including diabetic retinopathy and osteoblast differentiation (Zeng et al., 2023; Xie et al., 2023; Su et al., 2019). Notably, SIRT1 can directly deacetylate p53, thereby inhibiting its transcriptional activity, which in turn may influence the expression of SIRT1-regulating miRNAs, forming a potential feedback loop (Fu et al., 2022). Our previous study demonstrated a role for p53 in SIRT1 downregulation in OGD/R-injured HUVECs following PCA treatment (Cao S. et al., 2022). Furthermore, Li et al. reported that p53 inhibits miR-29b expression in human Tenon’s fibroblasts (Li et al., 2023). In the present study, we found that PCA- and NAC-induced overexpression of miR-29b-3p led to p53 downregulation, which in turn promoted SIRT1 activity and expression. Collectively, these findings support a model in which the ROS/miR-29b-3p/SIRT1 axis mediates the protective effects of PCA against OGD/R-induced endothelial injury by promoting autophagy and inhibiting apoptosis.

This study identifies miR-29b-3p as a key mediator of the cytoprotective effects of PCA, extending our previous work on SIRT1 (Cao S. et al., 2022). Mechanistically, our data reveal a reciprocal regulatory circuit between SIRT1 and miR-29b-3p, in which SIRT1 deacetylates and inactivates p53, thereby relieving p53-mediated repression of miR-29b-3p, while miR-29b-3p in turn enhances SIRT1 expression under OGD/R conditions, likely through indirect mechanisms potentially involving other direct targets of miR-29b-3p or a ceRNA network that override its direct 3′-UTR binding. This regulation enables PCA to simultaneously promote autophagy and inhibit apoptosis via the same signalling axis, amplifying its pleiotropic protective effects beyond what a simple linear pathway would predict. Given that endothelial dysfunction is a critical event in I/R injury, targeting the ROS/miR-29b-3p/SIRT1 regulatory network with PCA may represent a promising therapeutic strategy for ischemic cardiovascular diseases.

Several limitations of this study should be acknowledged. The primary caveat is that all experiments were conducted *in vitro* using HUVECs, and while OGD/R is a widely accepted model for simulating I/R injury, *in vivo* validation in animal models of cardiac or cerebral I/R injury will be essential to confirm the physiological relevance of our findings. Additionally, the dual-luciferase reporter assay was performed in HEK-293T cells rather than HUVECs due to their superior transfection efficiency. Although this is a standard approach for mechanistic reporter studies, verification of this direct interaction in HUVECs under OGD/R stress would further strengthen our conclusion. The 48-h wound-healing assay may also be confounded by proliferative effects in addition to cell migration, and future studies employing time-lapse imaging or Boyden chamber assays could more precisely dissect these two components. Finally, although our data establish a reciprocal feedback loop between SIRT1 and miR-29b-3p, the potential involvement of other miRNAs or downstream effectors cannot be excluded. Future unbiased screening approaches will be valuable to identify the direct targets through which miR-29b-3p indirectly modulates SIRT1 in this context.

## 5 Conclusion

In summary, this study demonstrates that PCA protects against OGD/R-induced endothelial injury by regulating miR-29b-3p through ROS suppression, which in turn enhances SIRT1 expression via p53-mediated indirect regulation, thereby promoting autophagy and inhibiting apoptosis. These findings identify PCA as a novel regulator of the ROS/miR-29b-3p/SIRT1 axis and suggest that PCA represents a promising lead compound for further *in vivo* investigation in ischemic cardiovascular diseases. Future studies are warranted to identify the direct targets through which miR-29b-3p indirectly modulates SIRT1 in this context.

## Data Availability

The original contributions presented in the study are included in the article/Supplementary Material, further inquiries can be directed to the corresponding authors.
