# Supplementary material for: Protocatechualdehyde ameliorates OGD/R-induced endothelial injury via the ROS/miR-29b-3p/SIRT1 regulatory axis
Source: Front Pharmacol. 2026 Aug 12;17:1904530. doi: 10.3389/fphar.2026.1904530 (PMC13506492; doi:10.3389/fphar.2026.1904530)
Supplement: Supplementary file 1 [file Supplementaryfile1.pdf]

Protocatechualdehyde alleviates OGD/R-induced endothelial cells injury through miR-29b-3p

Yanqing Wu[1+], Xilin Qiao[1+], Shuaiyu Chen[1+], Yalan Lu[1], Bingyan Mao[1], Mengdie Zhu[1], Xinzhi Chen[1], Nipi Chen[1], Zhishan Ding[2*], Bo Jin[1*]

[1]School of Life Science, Zhejiang Chinese Medical University, Hangzhou 310053, Zhejiang, China

[2]School of Medical Technology and Information Engineering, Zhejiang Chinese Medical University, Hangzhou 310053, Zhejiang, China

[+]these authors contributed equally to this work.

[*]Correspondence: jinbo@zcmu.edu.cn; dzszjtcm@163.com

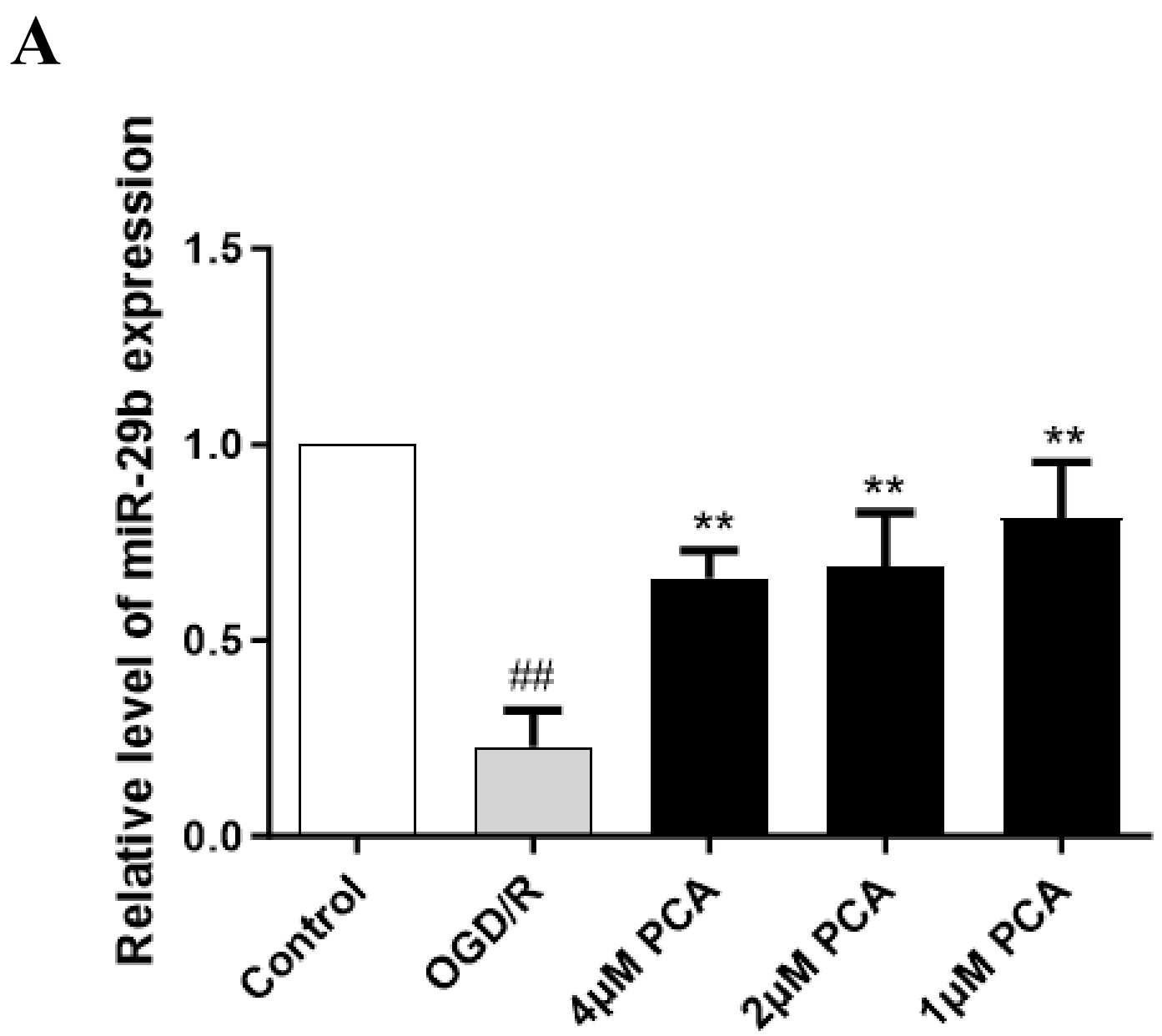

Figure 1A. HUVECs were induced with OGD/R, and treat with PCA for 24 h, and then miR-29b-3p expression was assessed by qRT-PCR. (##*P<0.01* vs Control, ***P<0.01* vs OGD/R)

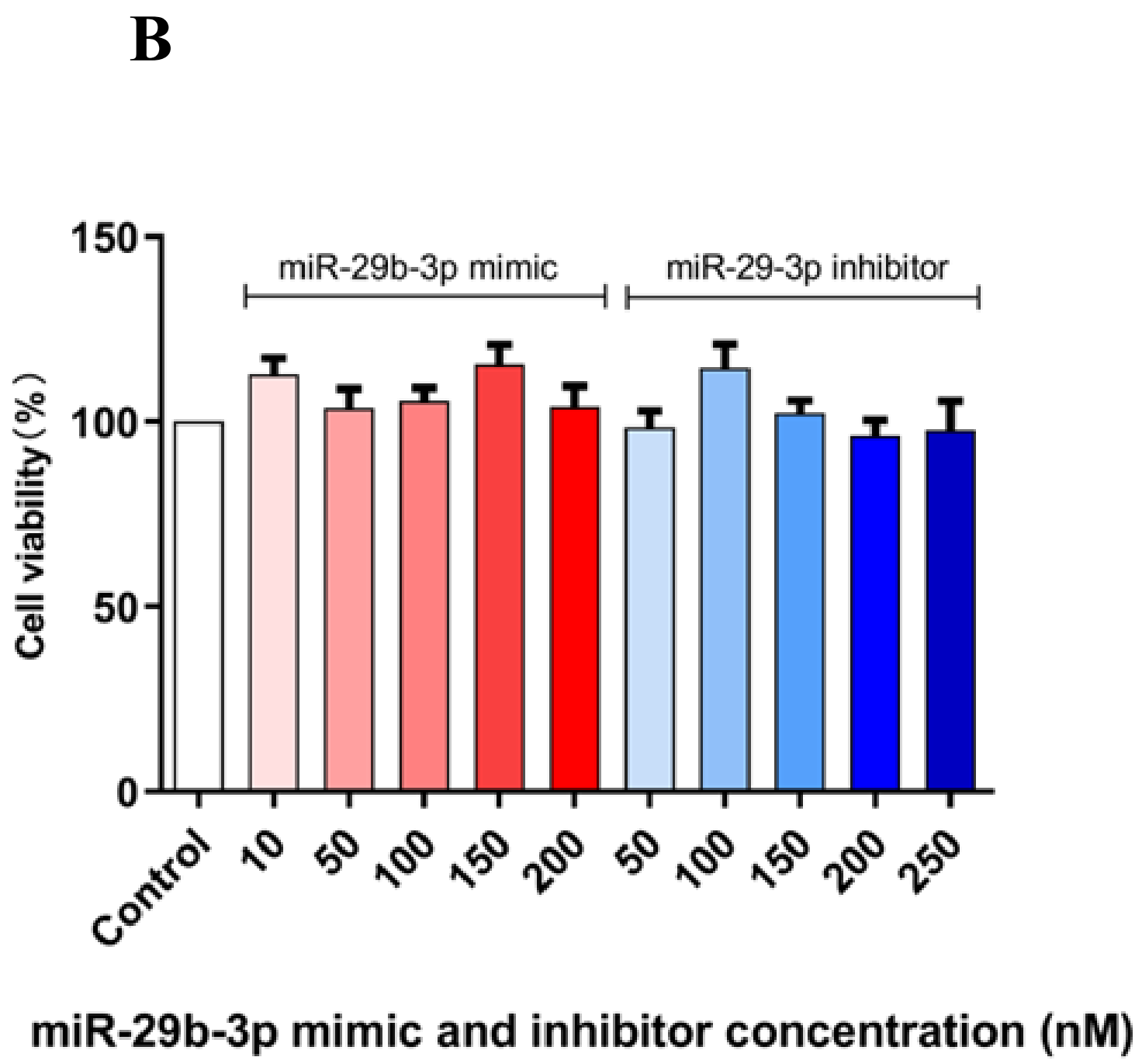

Figure 1B. Different concentrations of miR-29b-3p mimic (10–200 nM) and miR-29b-3p inhibitor (50–250 nM) were applied to culture for 6 h. A CCK-8 assay was used to evaluate cell viability.

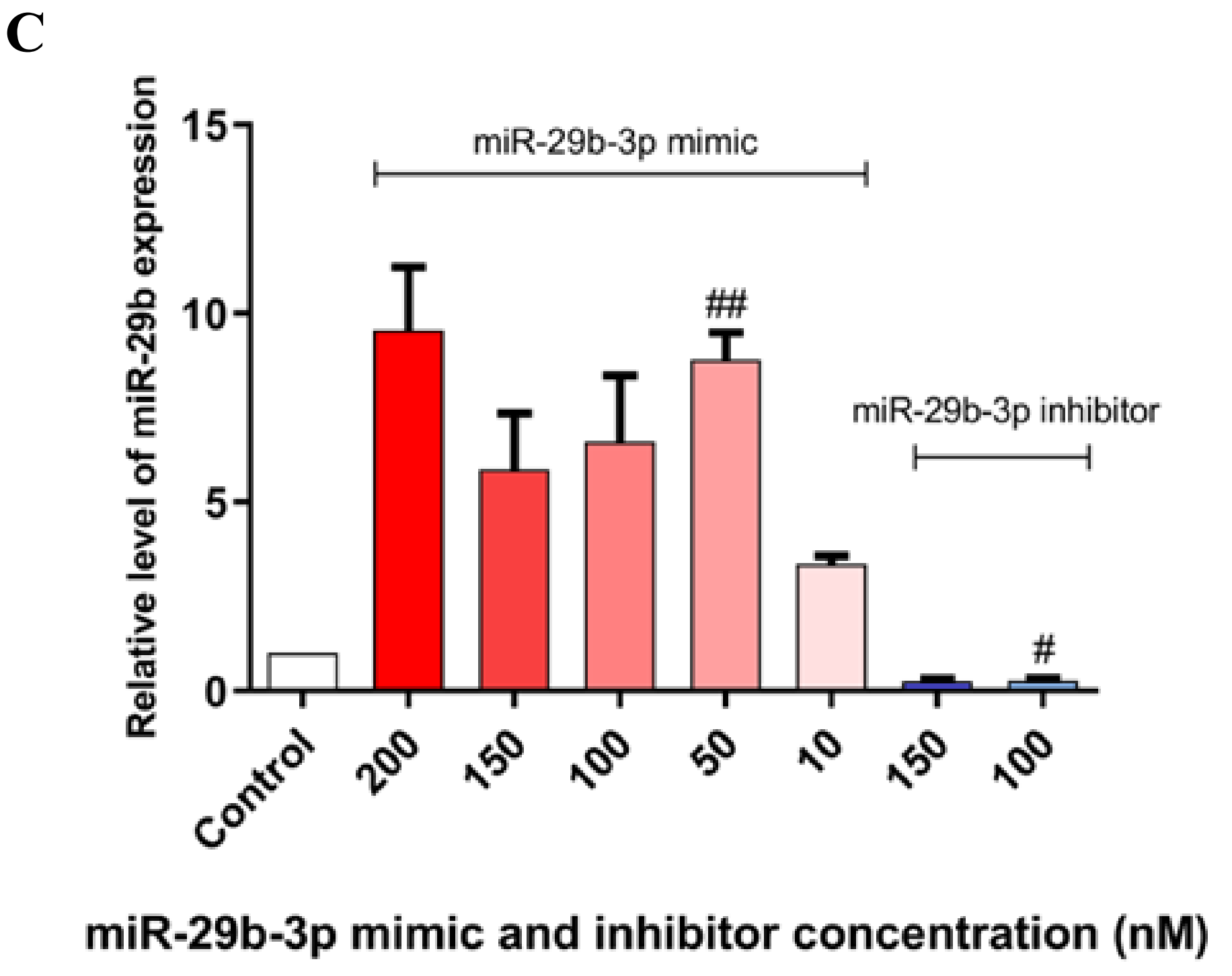

Figure 1C. Validation of transfection effects of different concentrations of miR-29b-3p mimic (10–200 nM) and miR-29b-3p inhibitor (100,150 nM) by real-time PCR. (*#P<0.05*, *##P<0.01* vs Control)

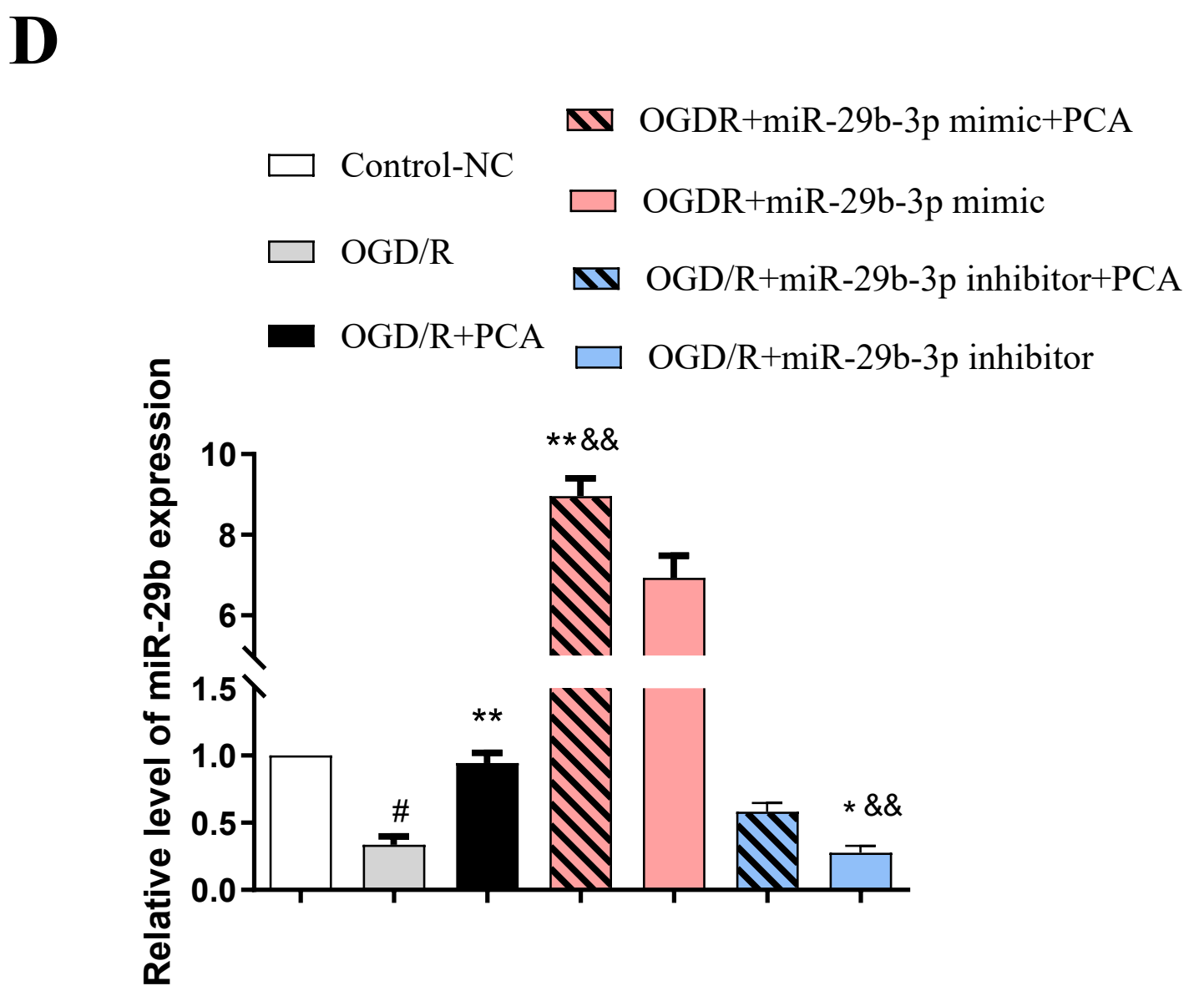

Figure 1D. Effect of miR-29b-3p mimic (50 nM) and miR-29b-3p inhibitor (100 nM) on the regulation of miR-29b-3p expression by PCA. (*##P<0.01* vs Control, **P<0.05*, ***P<0.01* vs OGD/R, *&&P<0.01* vs 1µM PCA+OGD/R)

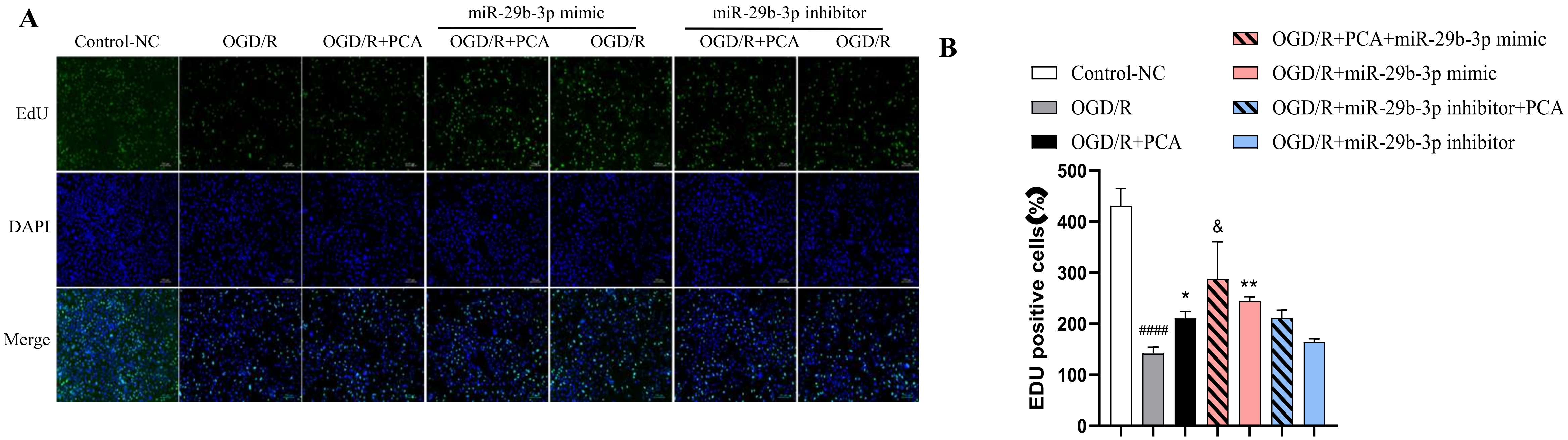

Figure 2A,B. Proliferation assay to evaluate the proliferative capacity of different cell groups.Scale bar=100 μm. (A) PCA promotes the proliferative ability of HUVECs through miR-29b-3p in OGD/R-induced injury. (B) $^{\#\#\#\#}$P<0.0001 vs. control, *P<0.05, **P<0.01, $^{\&}$P<0.05 vs. 1 μM PCA +OGD/R.

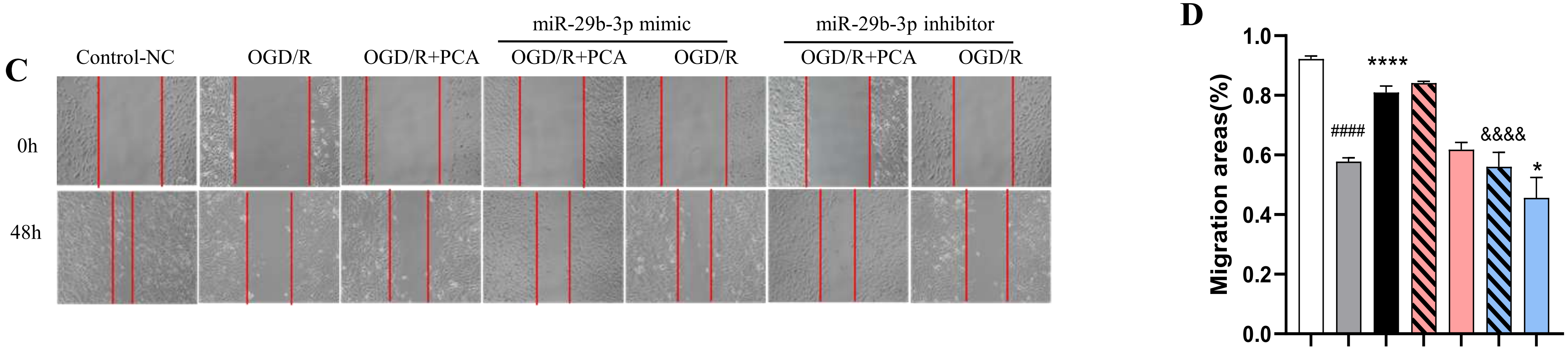

Figure 2C,D. **Scratch assay to assess the migration ability of each cell group. Scale bar=100 µm. (C) PCA promotes the migratory ability of HUVECs through miR-29b-3p. $^{####}$P<0.0001 vs. control, *P<0.05, **P<0.01, ****P<0.0001 vs. OGD/R, $^{\&\&\&\&}$P<0.0001 vs. 1 µM PCA +OGD/R.**

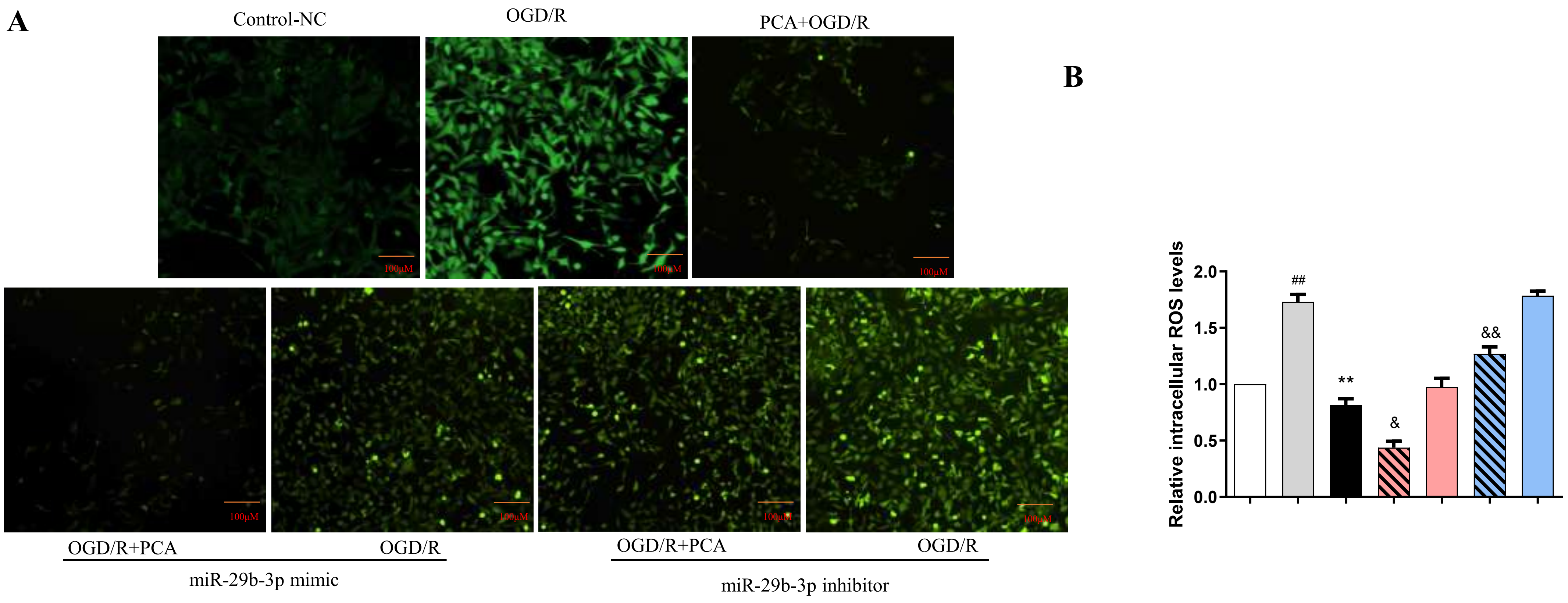

C

Figure 3A,B. Representative images of ROS stained with DCFH-DA in HUVECs were obtained by a fluorescent microscopy. Scale bar = 100 μm. ROS analysis was performed for intracellular ROS level. (##*p<0.01* vs Control，*P<0.01* vs OGD/R，&*P<0.05*, &&*P<0.01* vs 1 μM PCA+OGD/R)**

C

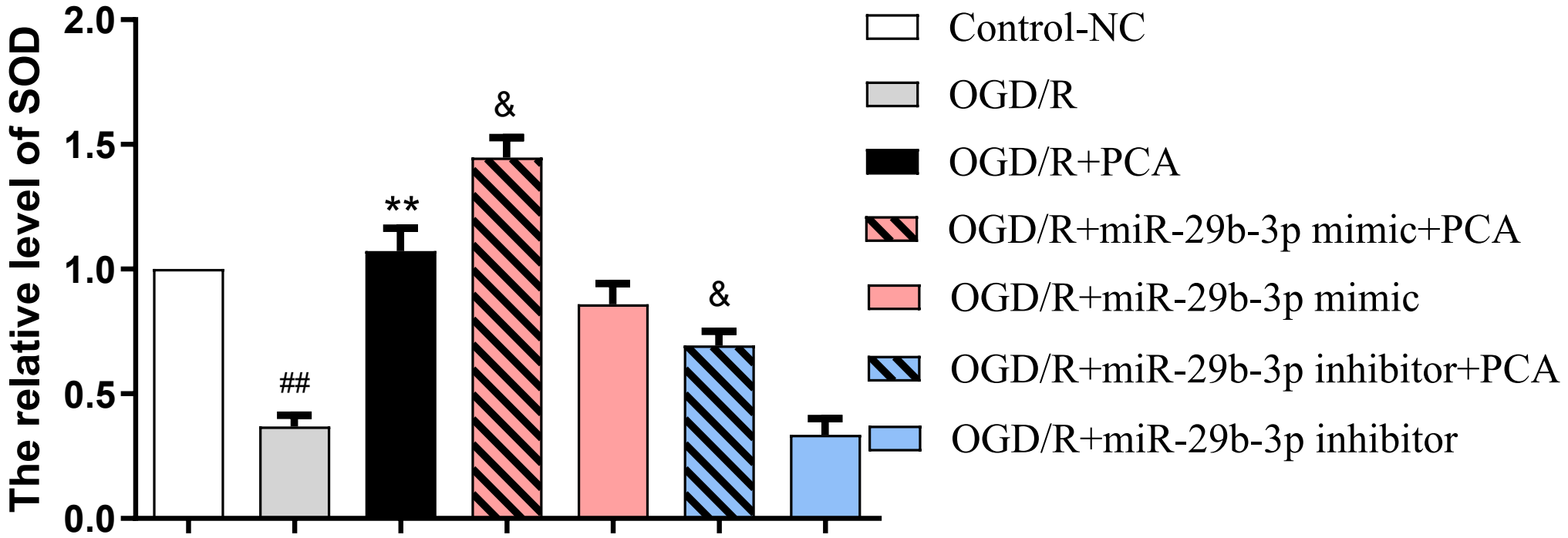

Figure 3C. The SOD activity was detected by total SOD assay kit. (*$^{\#\#}P<0.01$* vs Control， *$^{**}P<0.01$* vs OGD/R，*$^{\&}P<0.05$* vs 1 μM PCA+OGD/R)

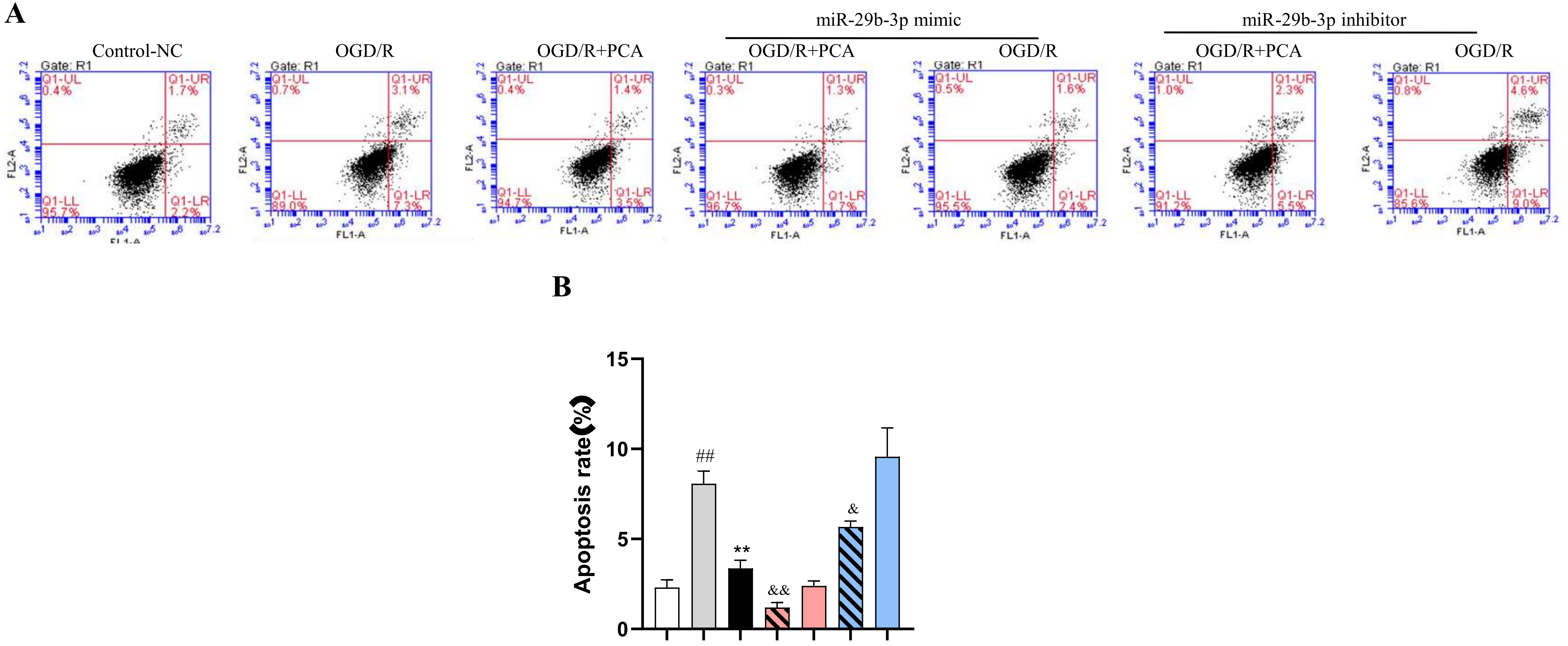

Figure 4A,B. Cell apoptosis was analyed by flow cytometry based on Annexin V-FITC and PI double staining. (##*P<0.01* vs Control，*P<0.01* vs OGD/R，&*P<0.05*, &&*P<0.01* vs 1 μM PCA+OGD/R)**

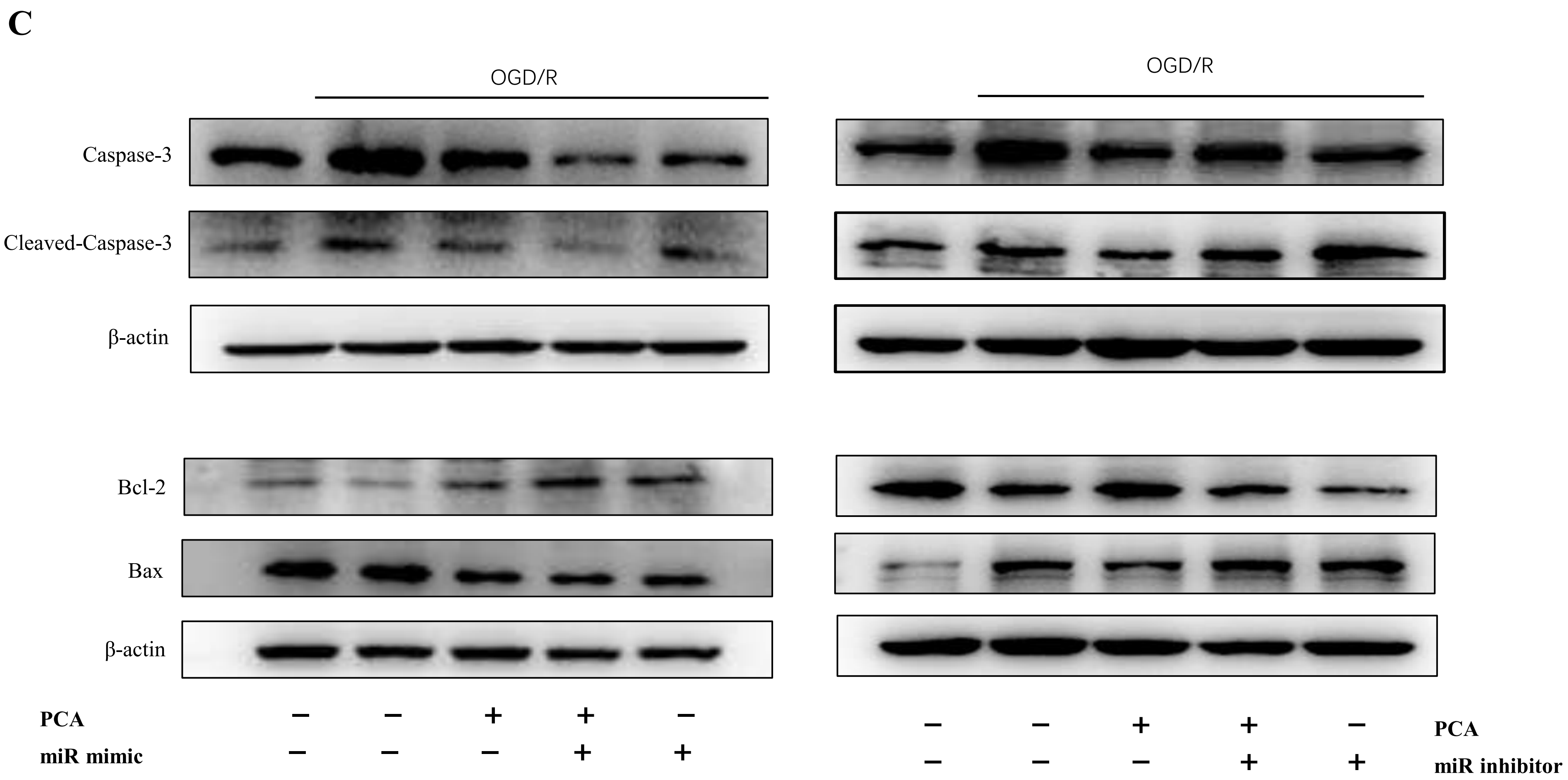

Figure 4C. Apoptosis-related proteins of Bax, Bcl-2, caspase-3 and cleaved caspase-3 were examined by western blotting. β-Actin was used as a loading control.

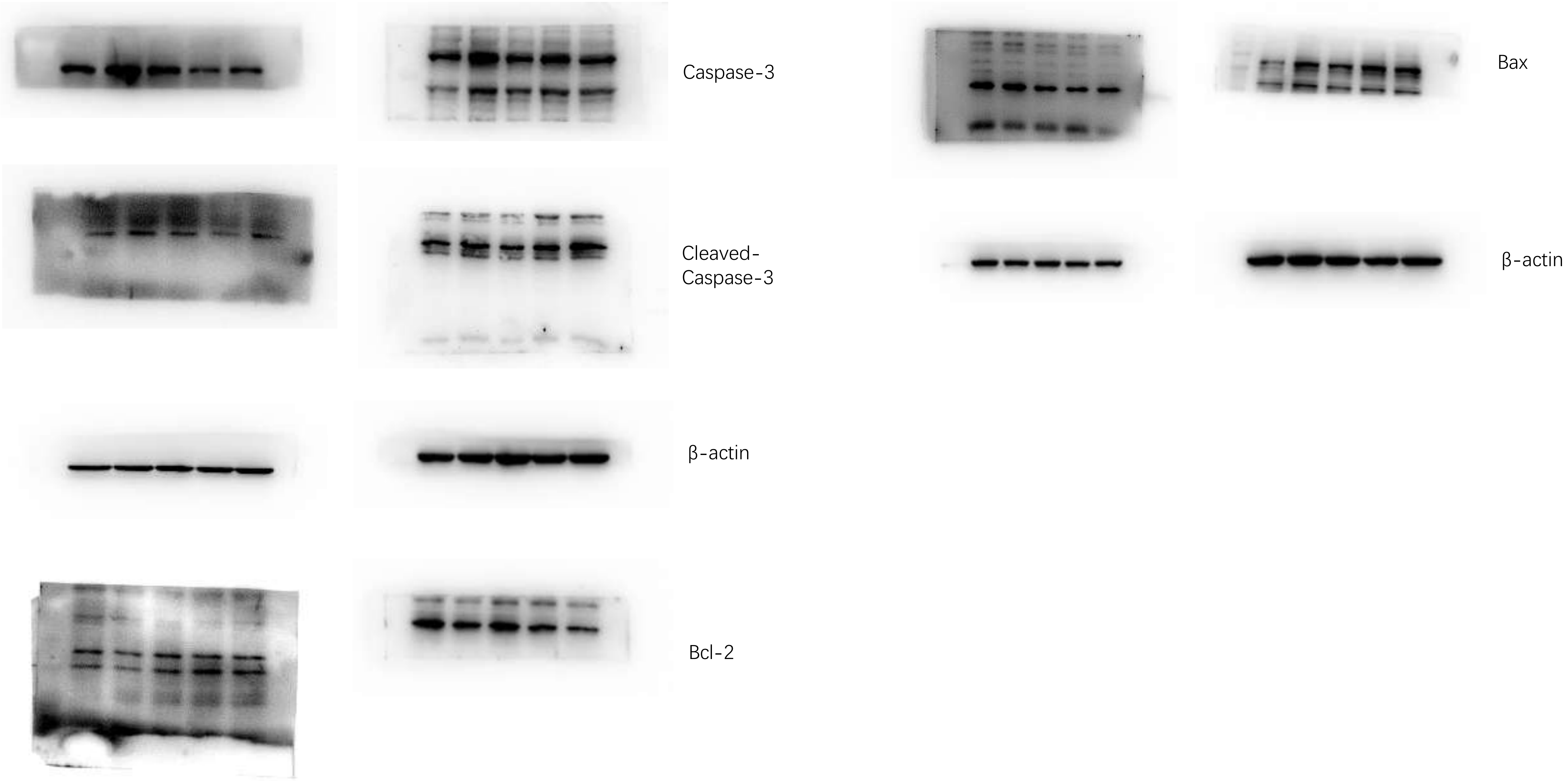
Caspase-3
Cleaved-
Caspase-3
β-actin
Bcl-2
Bax
β-actin

D

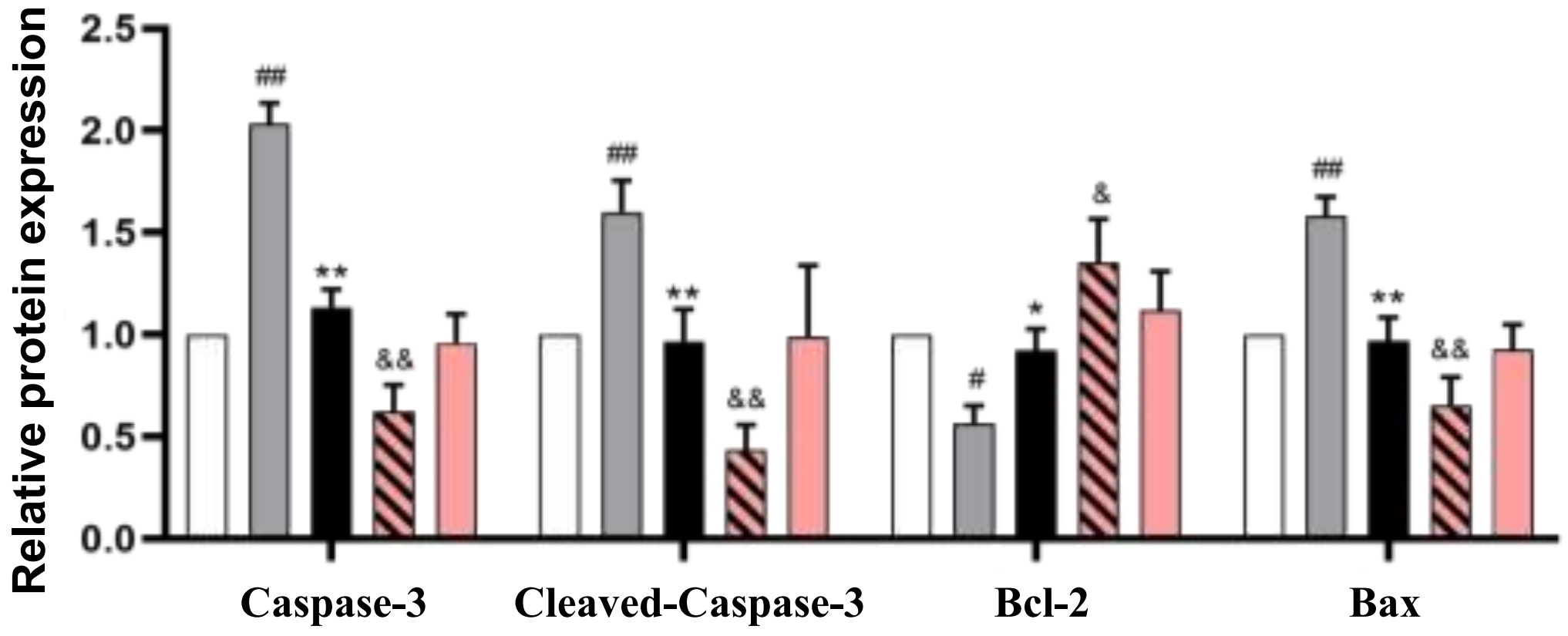

Figure 4D. Bax, Bcl-2, caspase-3, cleaved caspase-3 protein expression (#*P<0.05*, ##*P<0.01* vs Control, **P<0.05*, ***P<0.01* vs OGD/R, &*P<0.05*, &&*P<0.01* vs 0.72 μM PCA+OGD/R)

E

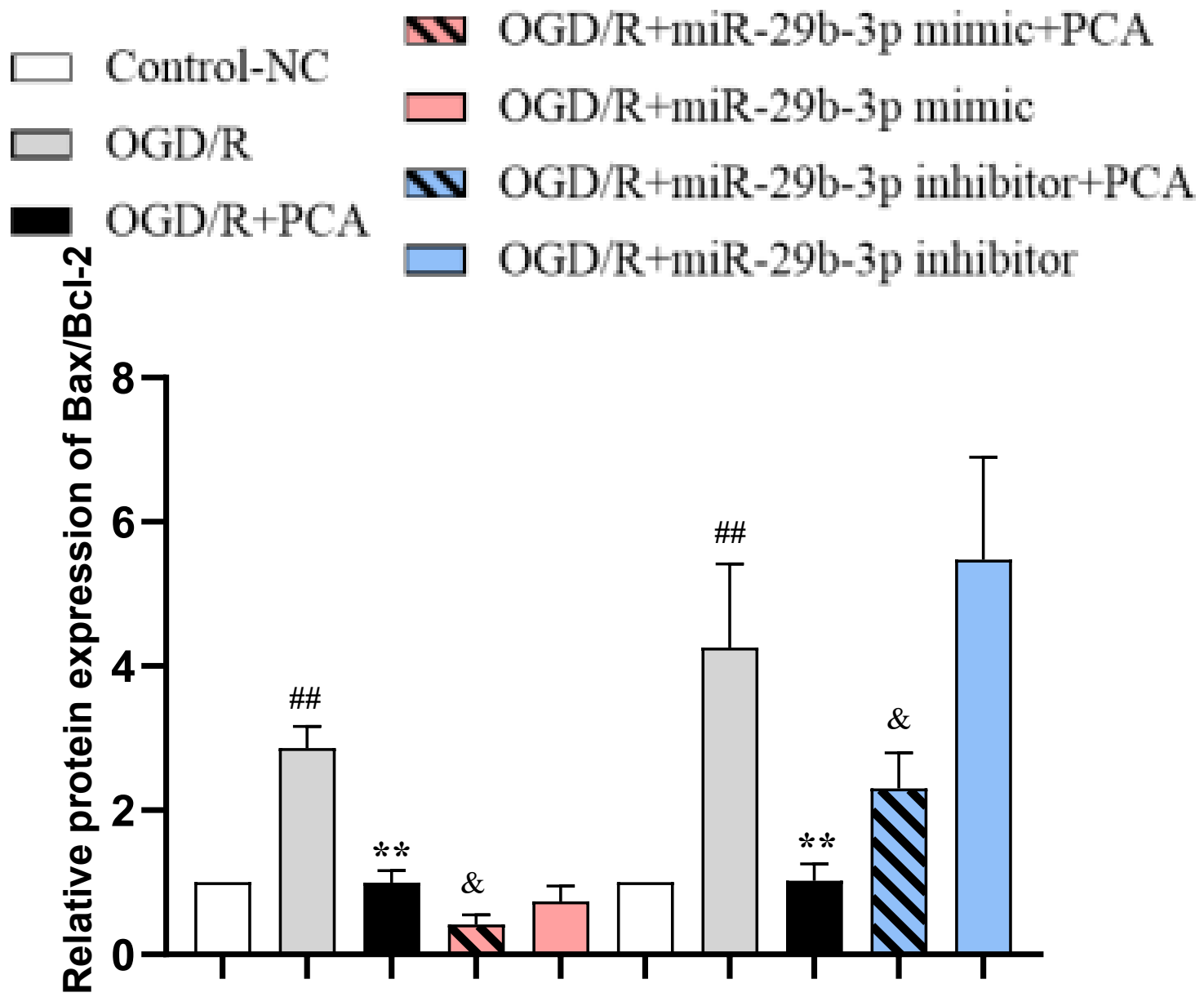

Figure 4E Bax/Bcl-2 ratio were quantified by densitometry and calculated with reference to β-Actin. (##*P<0.01* vs Control, ***P<0.01* vs OGD/R, &*P<0.05*vs 1 μM PCA+OGD/R)

F

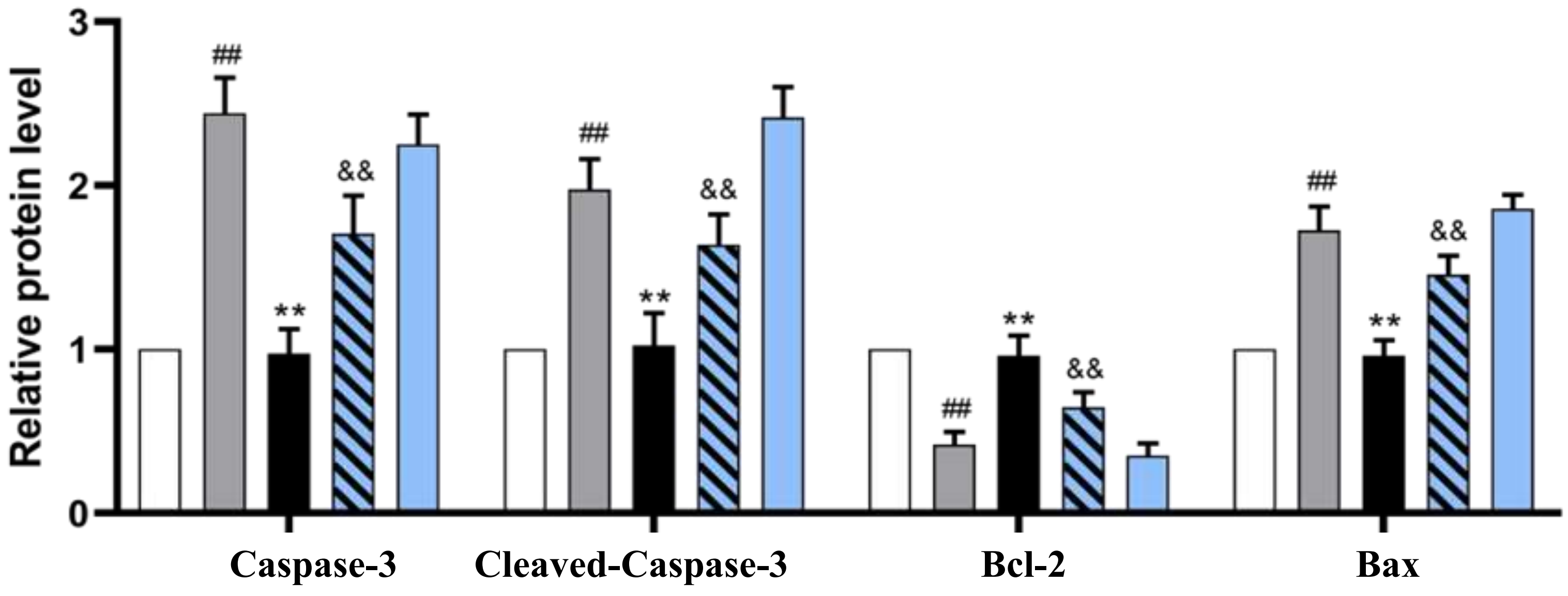

Figure 4F. Bax, Bcl-2, caspase-3, cleaved caspase-3 protein expression were quantified by densitometry and calculated with reference to β-Actin. (#*P<0.05*, ##*P<0.01* vs Control, **P<0.05*, ***P<0.01* vs OGD/R, &*P<0.05*, &&*P<0.01* vs 1 μM PCA+OGD/R)

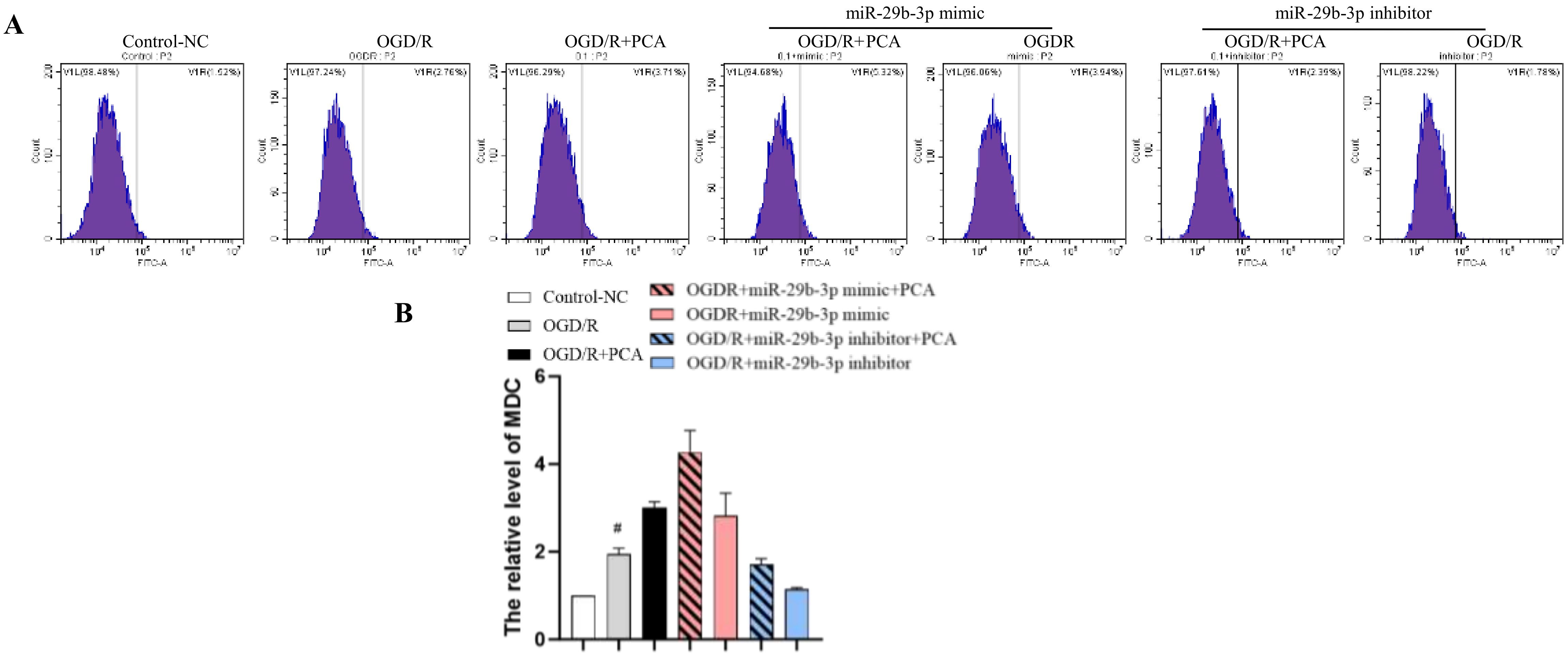

Figure 5A,B. The expression of MDC determined by flow cytometry. (#*P<0.05* vs Control)

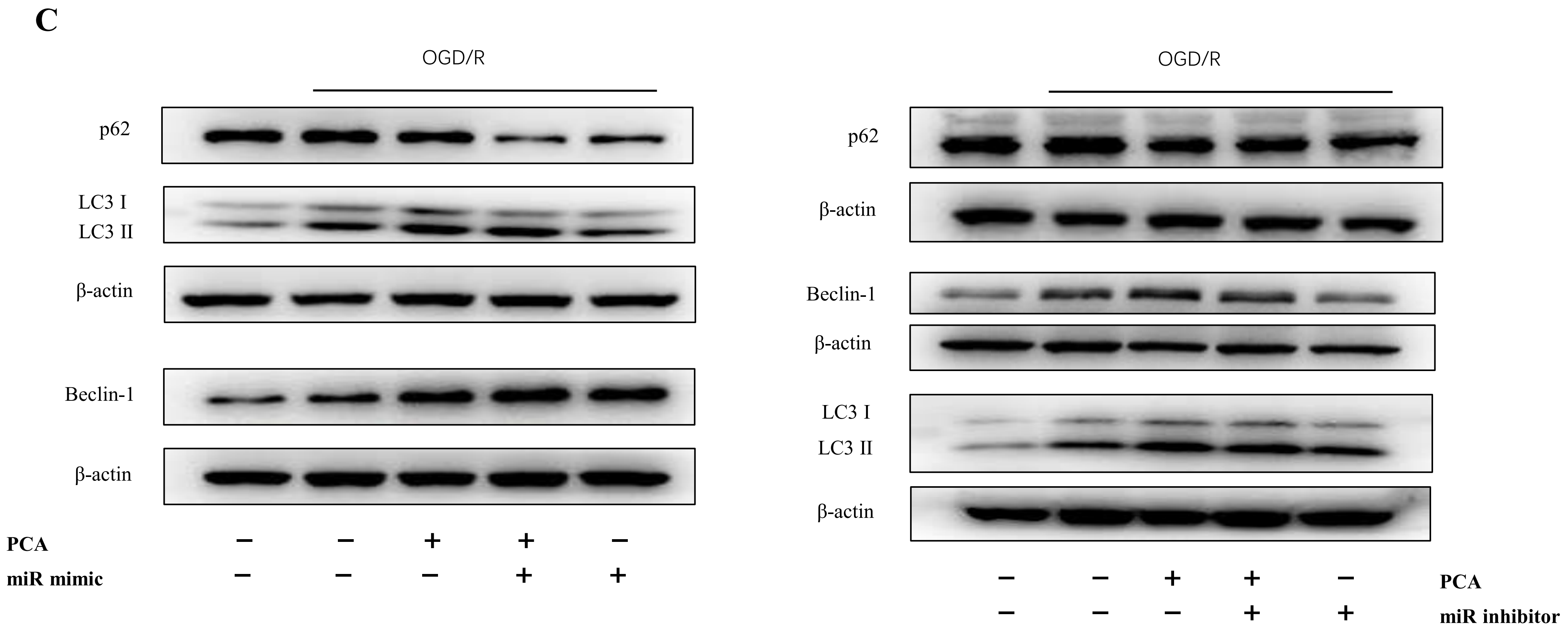

Figure 5C. Autophagy related proteins of p62、Beclin1 and LC3 were examined by western blotting. β-Actin was used as a loading control.

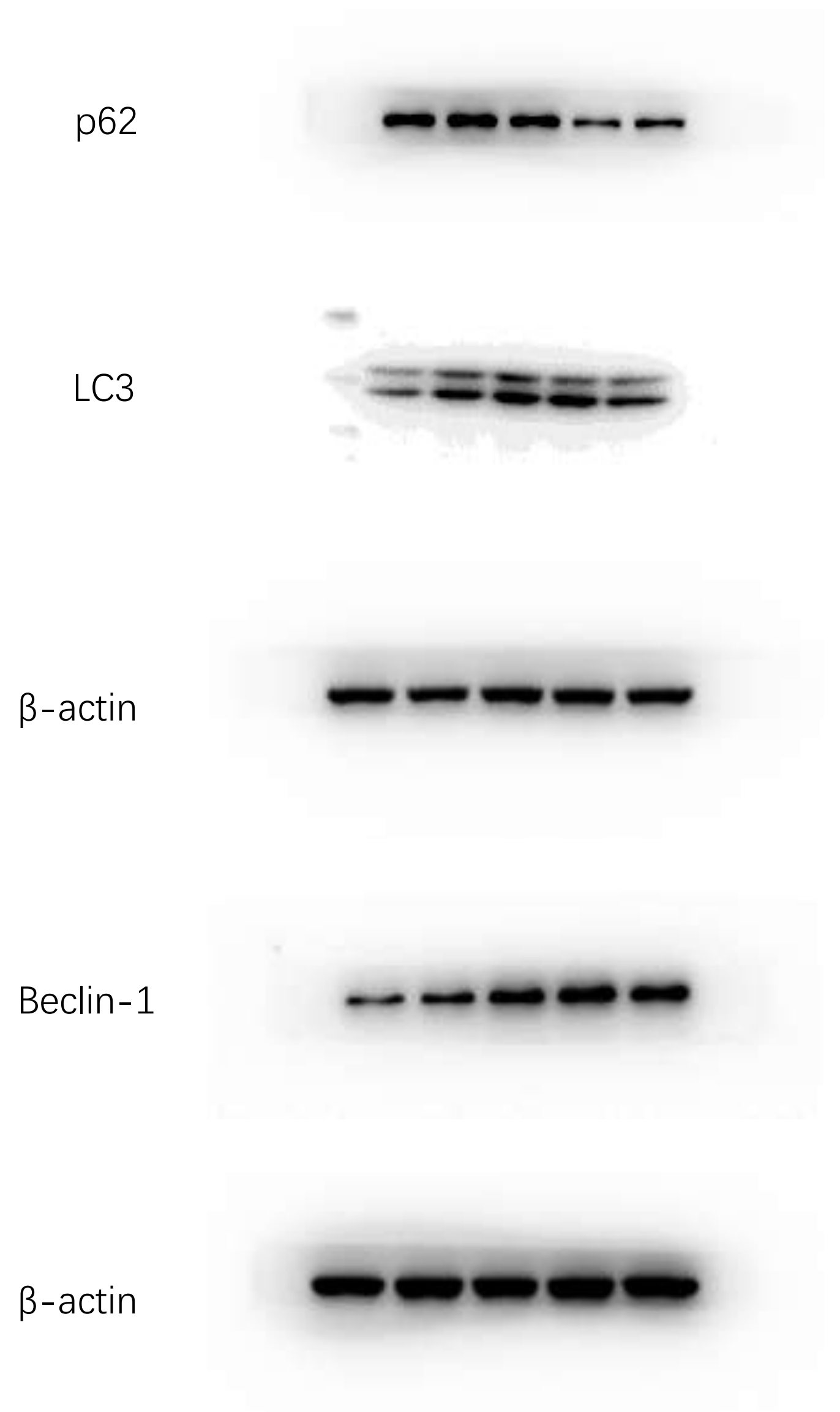
p62
LC3
β-actin
Beclin-1
β-actin

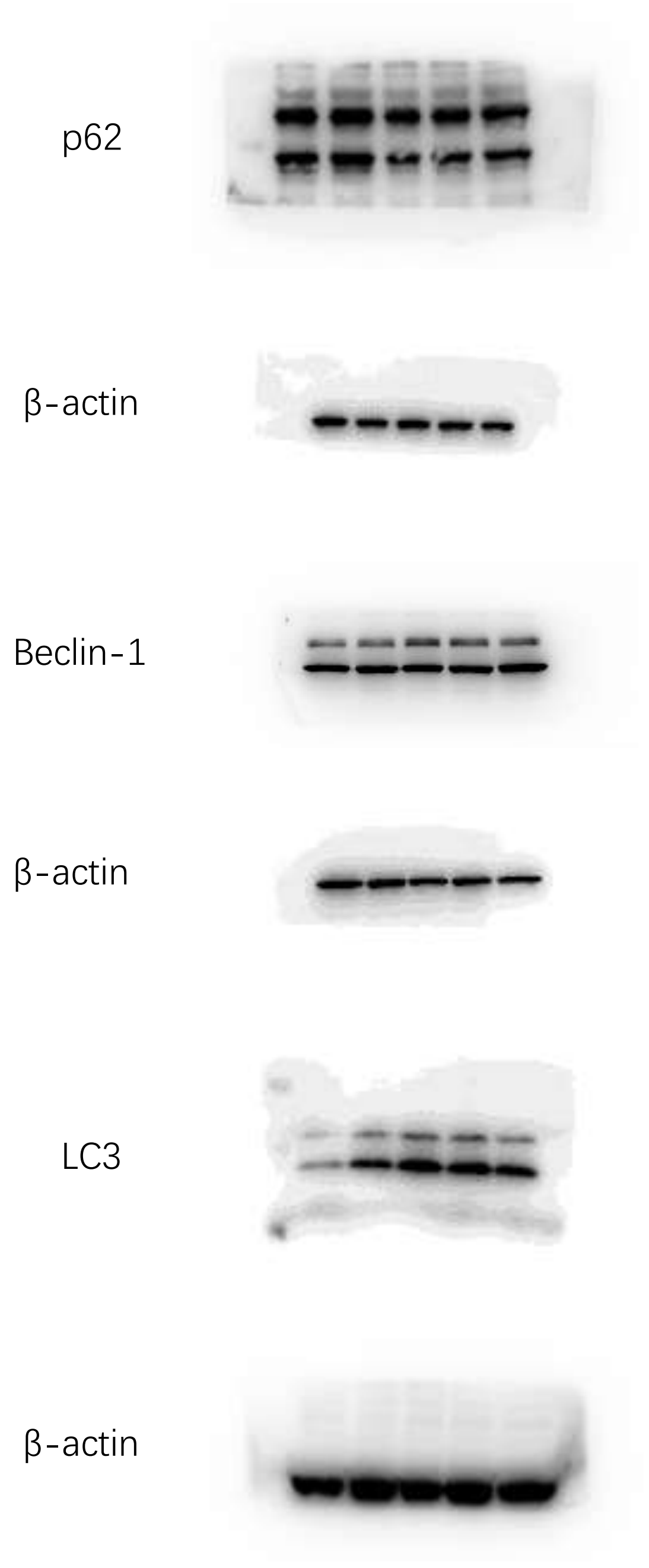
p62
β-actin
Beclin-1
β-actin
LC3
β-actin

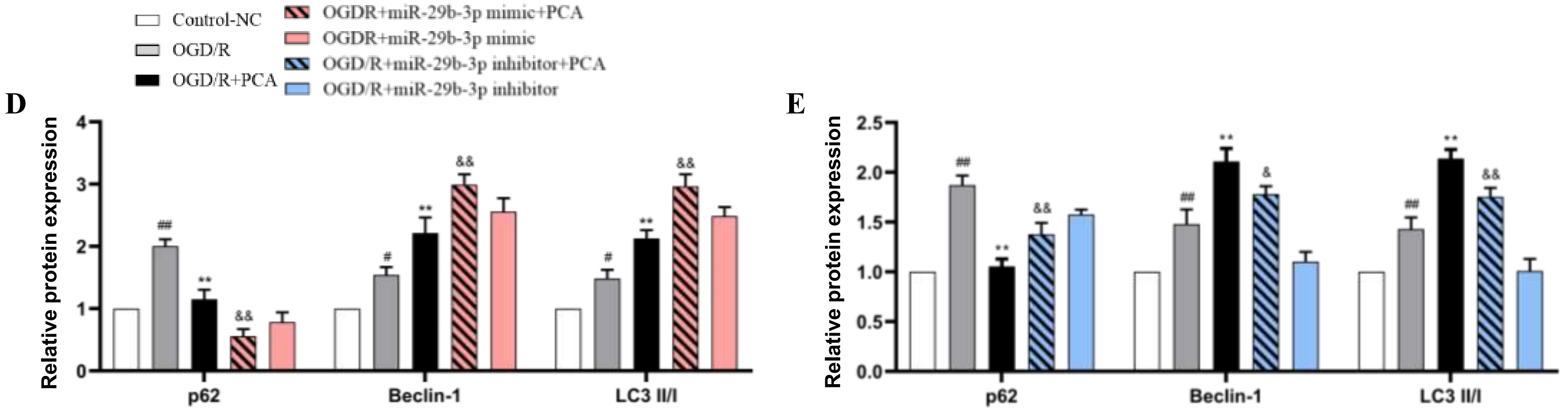

Figure 5D,E. Densitometric analysis of p62 and Beclin1 protein expression based on western blot assays. LC3 II/I ratio quantified by densitometry and calculated with reference to β-Actin. ($^{\#}$*P<0.05*, $^{\#\#}$*P<0.01* vs Control, **P<0.05*, ***P<0.01* vs OGD/R, $^{\&\&}$*P<0.01* vs 1 μM PCA+OGD/R)

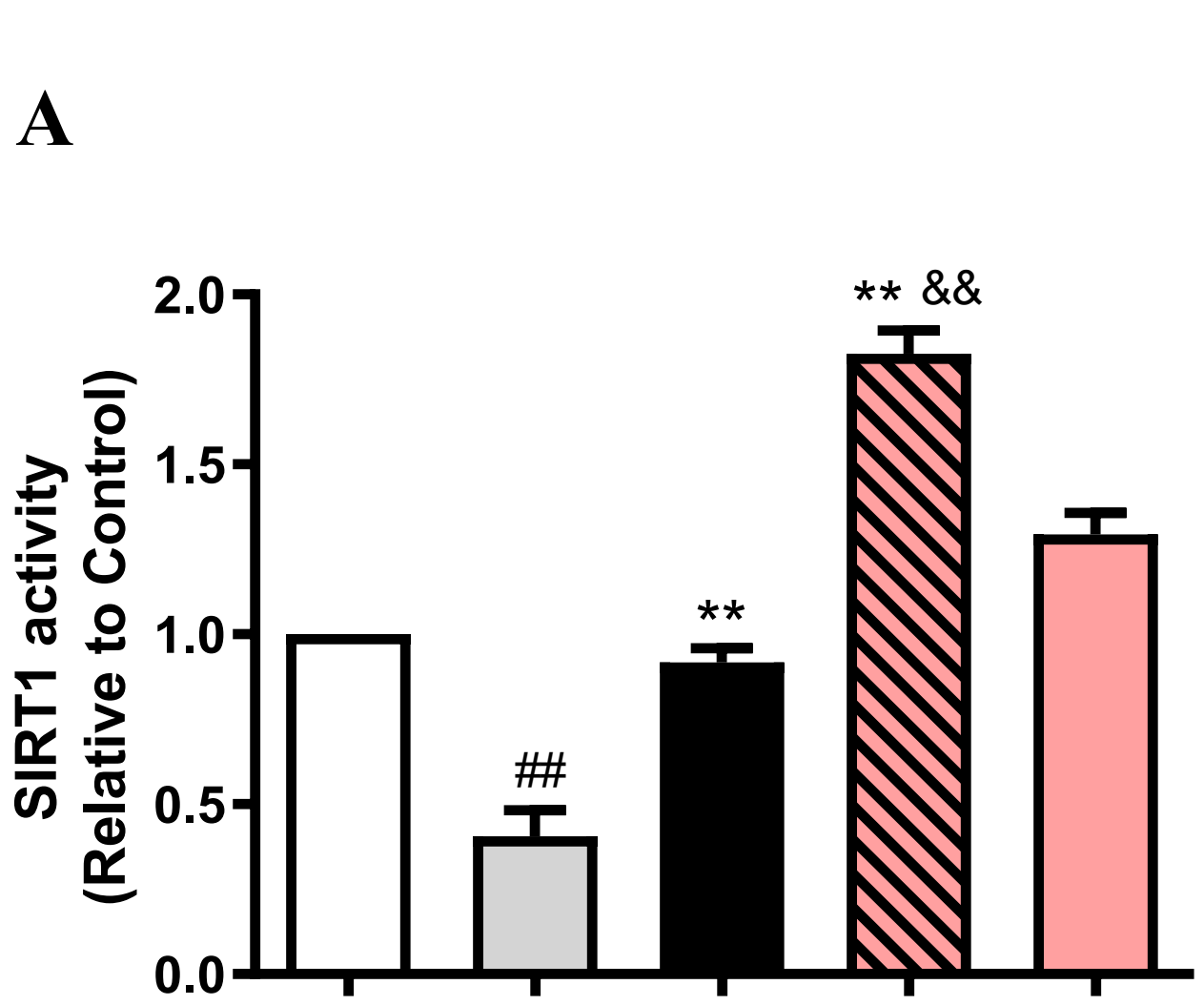

Figure 6A,D. SIRT1 activity was measured by an enzyme-linked immunosorbent assay kit. (##*P<0.01* vs Control, **P<0.05*, ***P<0.01* vs OGD/R, &*P<0.05*, &&*P<0.01* vs 1 μM PCA+OGD/R)

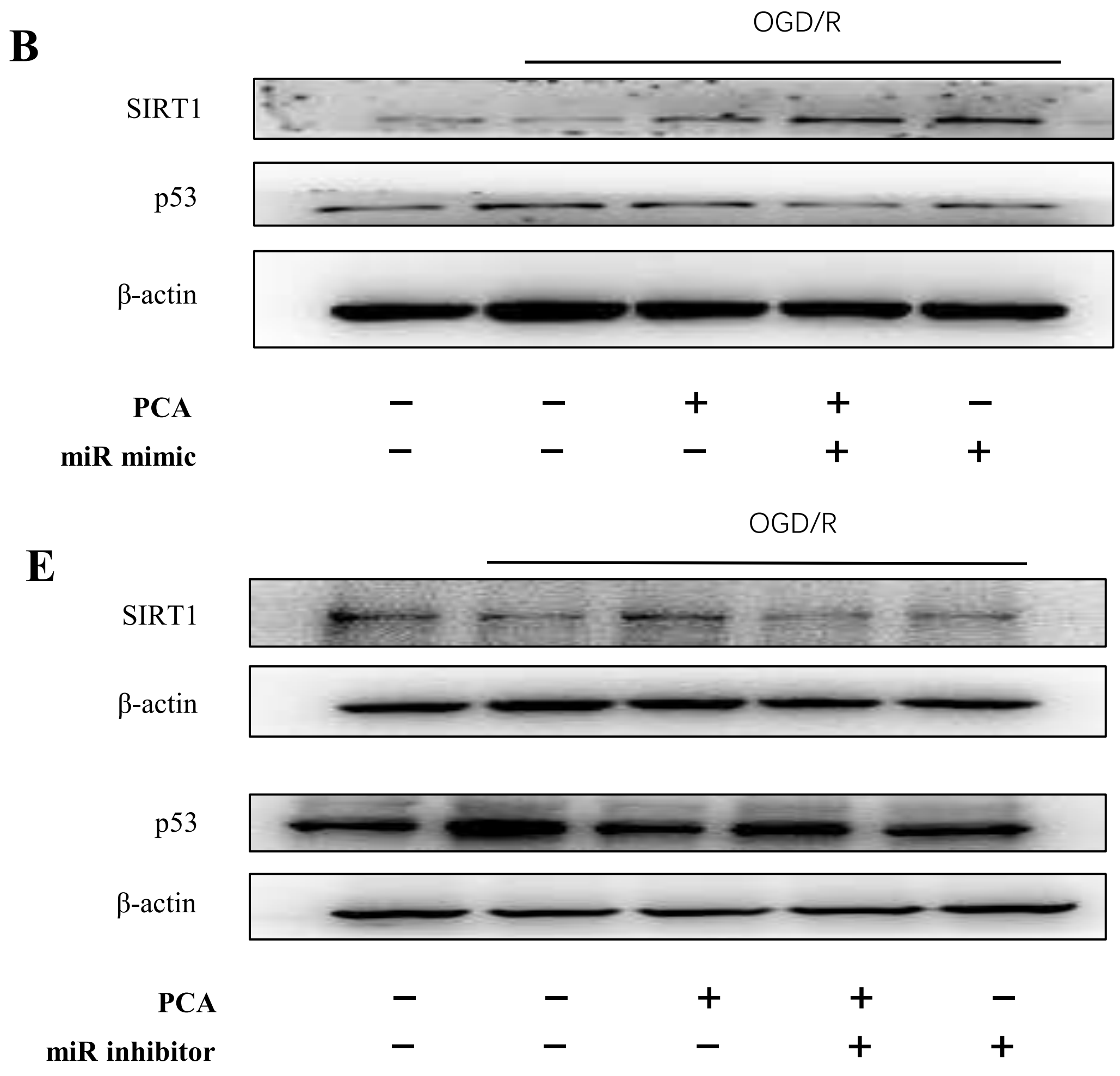

Figure 6B,6E. Determination of SIRT1 and p53 protein expression by western blotting. β-Actin was used as a loading control.

SIRT1

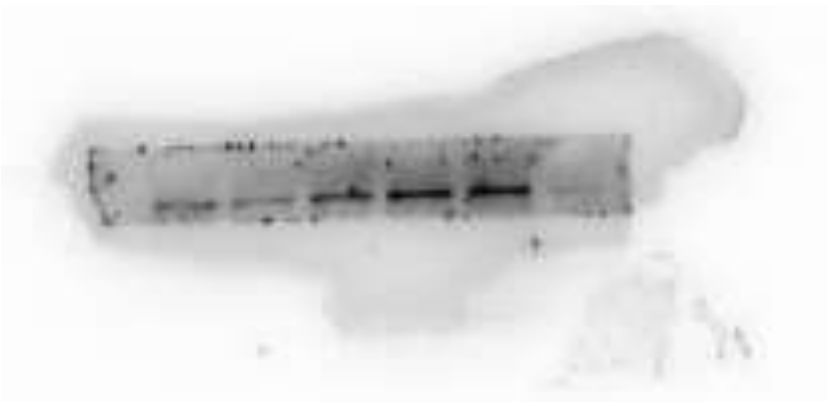

p53

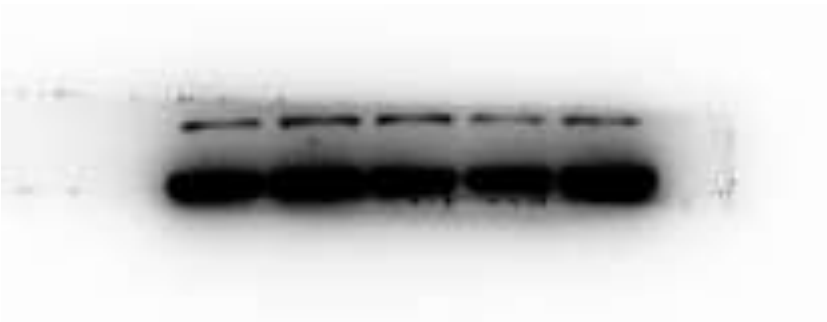

β-actin

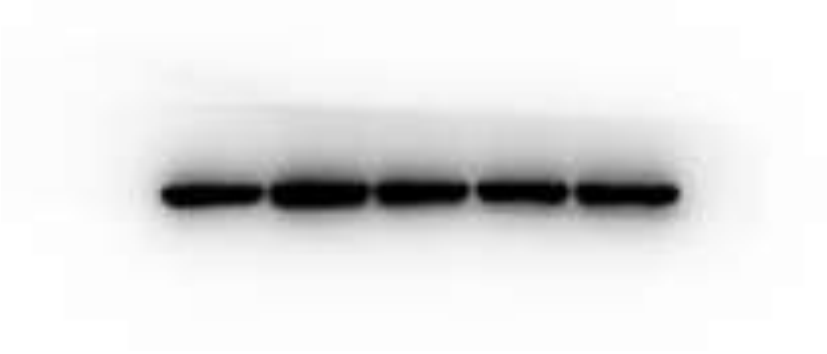

SIRT1

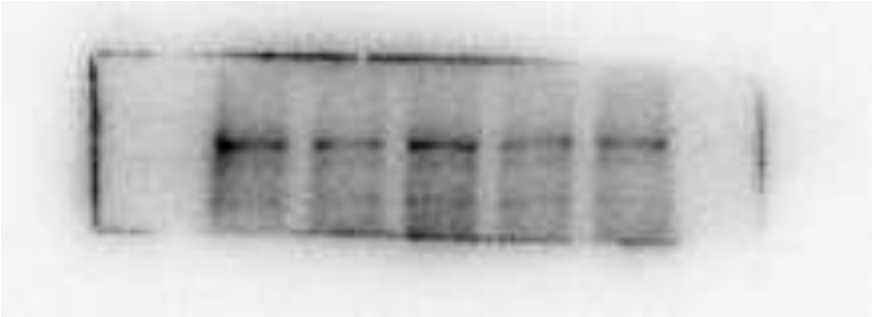

β-actin

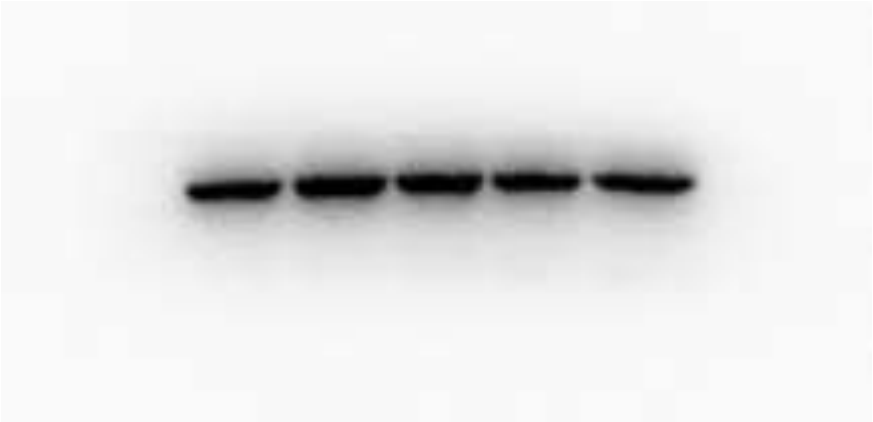

p53

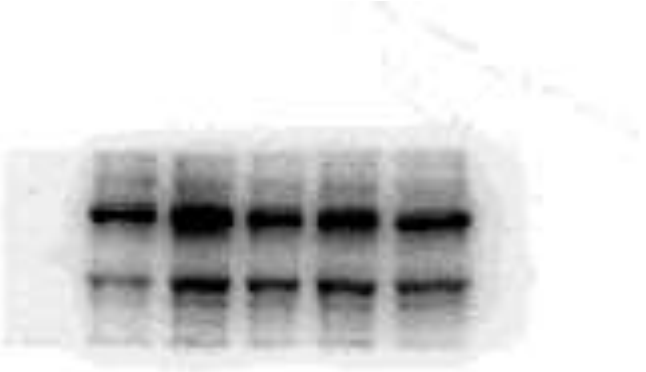

β-actin

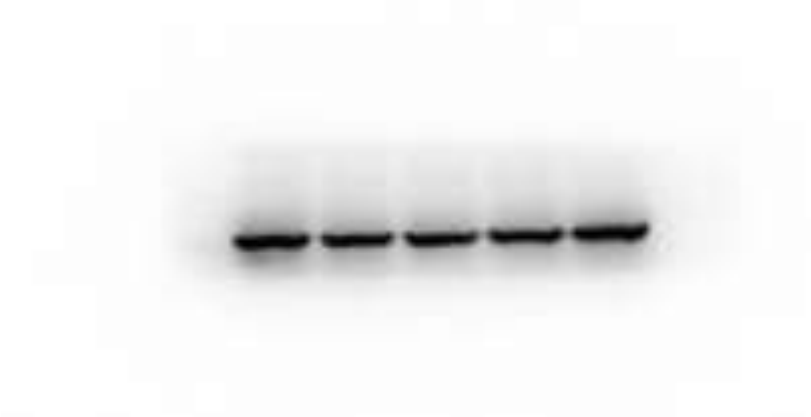

C

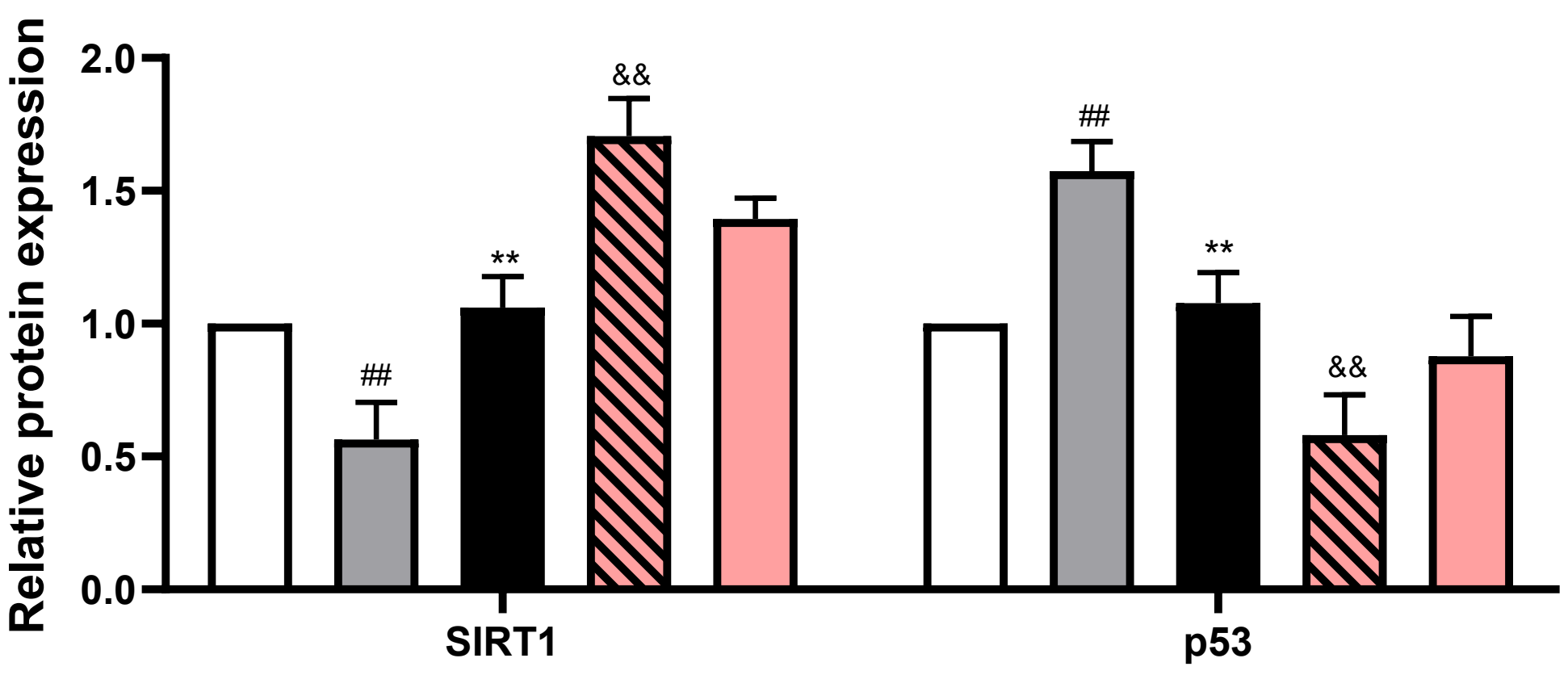

F

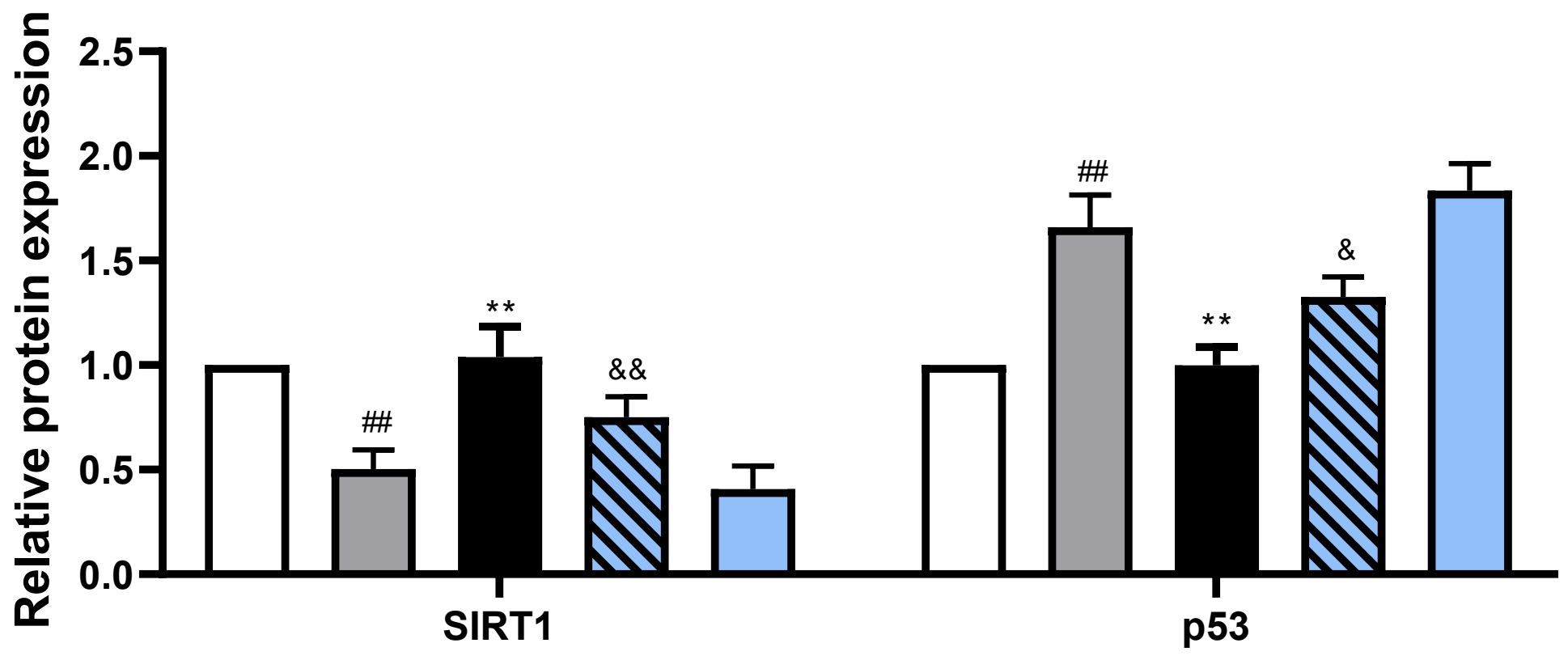

Figure 6C,6F. Densitometric analysis of SIRT1 and p53 protein expression based on western blot assays.
($^{\#\#}$*P<0.01* vs Control, **P<0.05*, ***P<0.01* vs OGD/R, $^{\&}$*P<0.05*, $^{\&\&}$*P<0.01* vs 1 μM PCA+OGD/R)

G

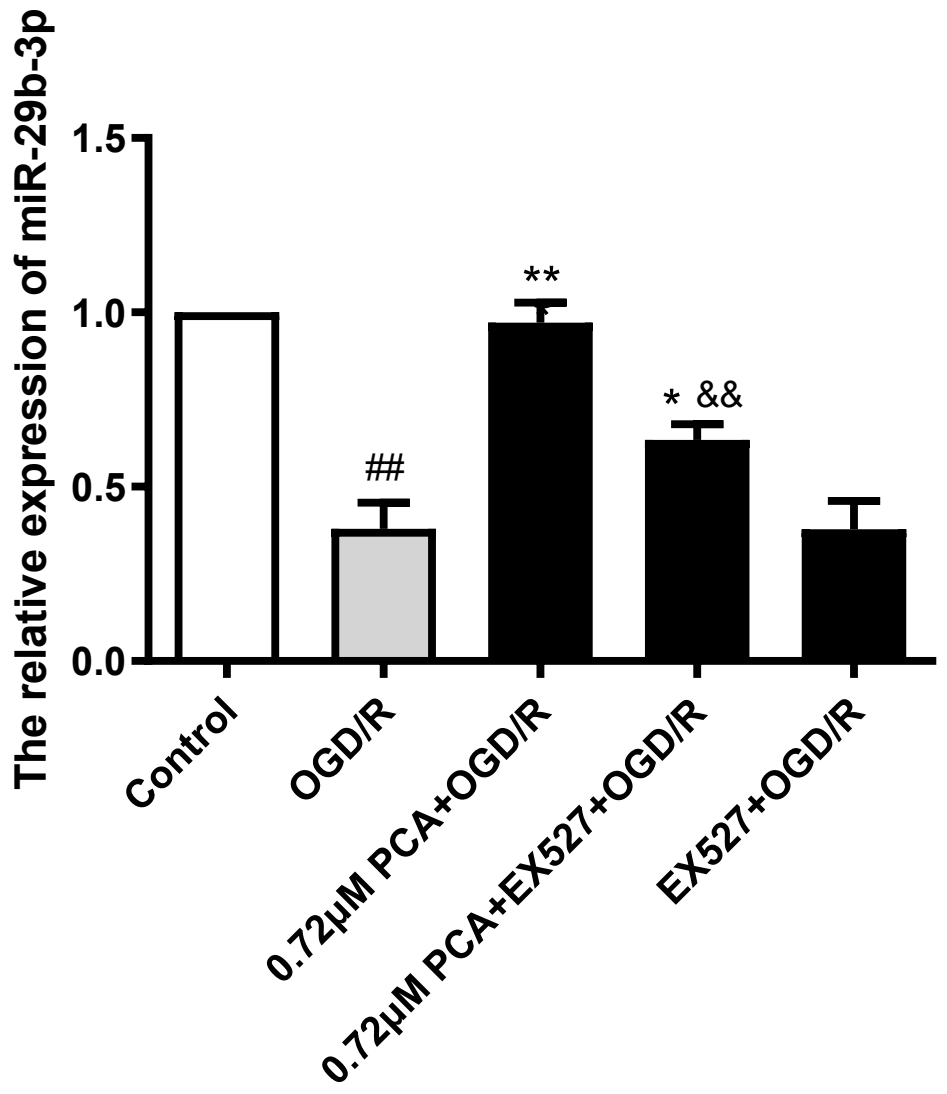

Figure 6G. Effect of EX527 inhibition of SIRT1 on miR-29b-3p expression in OGD/R-induced injury in HUVECs. ($^{\#\#}$*P<0.01* vs Control, **P<0.05*, ***P<0.01* vs OGD/R, $^{\&\&}$*P<0.01* vs 1 μM PCA+OGD/R)

H

Position 635-642 of SIRT1 3'UTR 5'- UUUAAAAGUAAUACUUGGUGCUA -3'

| | | | | | | |

hsa-miR-29b-3p 3'- UUGUGACUAAAGUUUACCACGAU -5'

I

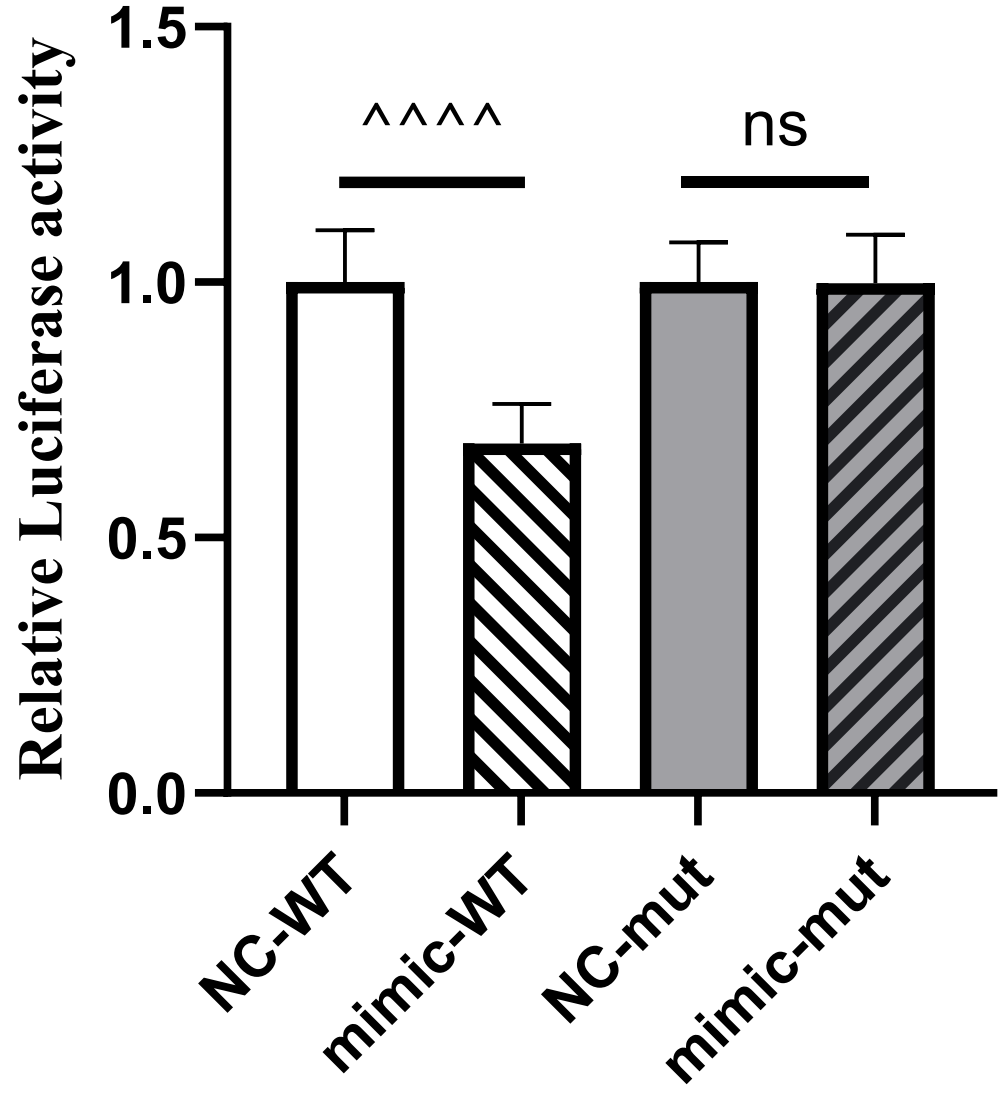

Figure 6H. Schematic diagram of the predicted miR-29b-3p binding site within the wild-type (WT) 3′-UTR of SIRT1 and the corresponding mutant (MUT) sequence. Figure 6I. Dual-luciferase reporter assay in HEK-293T cells co-transfected with miR-29b-3p mimic or NC, together with firefly luciferase reporters carrying either the WT or MUT 3′-UTR of SIRT1, and a Renilla luciferase plasmid for normalization. Luciferase activities were measured 48 h post-transfection. ^^^^$P < 0.0001$ vs NC-WT.

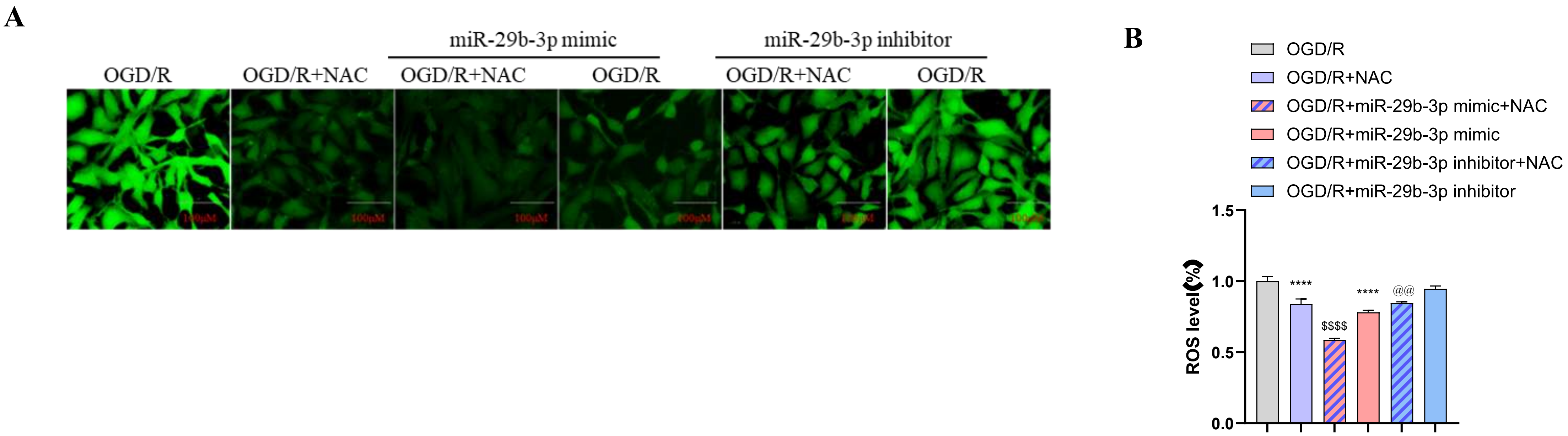

Figure 7A,7B. Representative images of ROS stained with DCFH-DA in HUVECs were obtained by a fluorescent microscopy. Scale bar=100 μm. NAC regulates the anti-apoptotic effect of miR-29b-3p in OGD/R-induced HUVECs. ****P<0.0001 VS. OGD/R, $^{\$\$\$\$}$P<0.0001 vs. OGD/R+mimic, $^{@@}$P<0.01 vs. OGD/R+inhibitor.

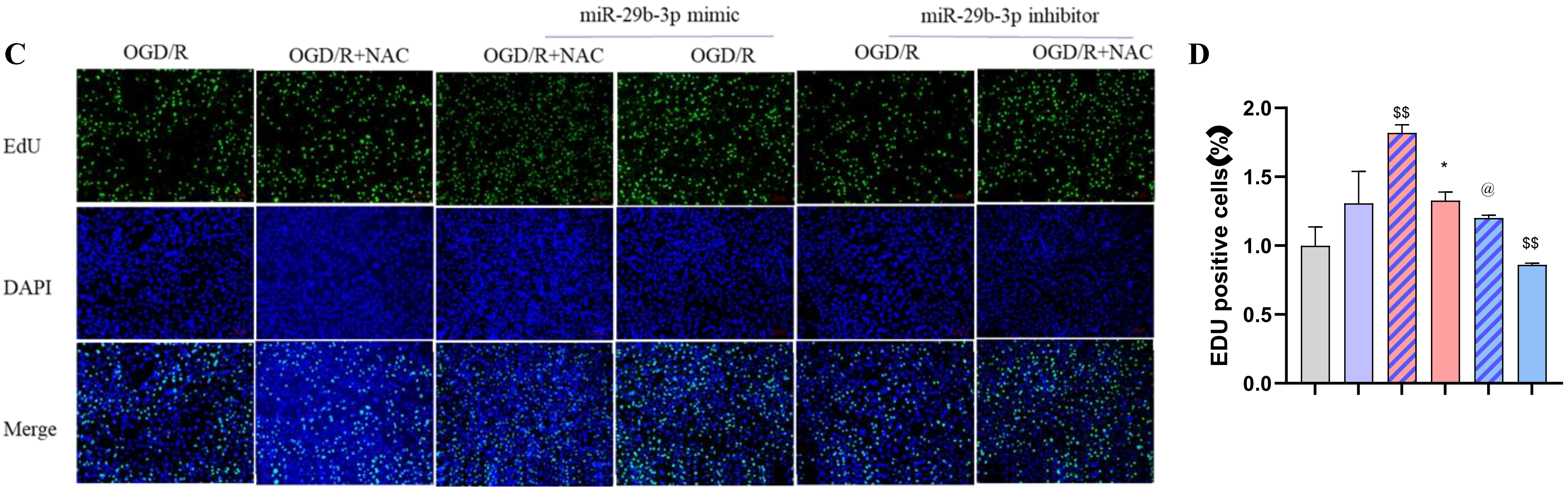

Figure 7C, 7D. Proliferation assay to evaluate the proliferative capacity of different cell groups.Scale bar=100 μm. NAC promotes the proliferative ability of HUVECs through miR-29b-3p in OGD/R-induc ed injury. *P<0.05 vs. OGD/R, $$P<0.01 vs. OGD/R+miR-29b-3p mimic，@P<0.05 vs. OGD/R+miR-29b-3p inhibitor.

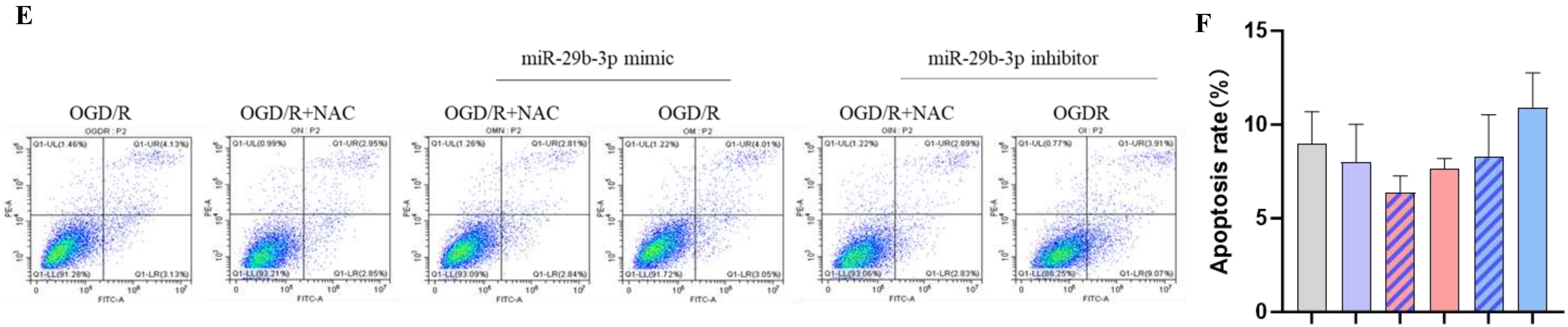

Figure 7E, 7F. NAC regulates the anti-apoptotic effect of miR-29b-3p in OGD/R-induced HUVECs.

G

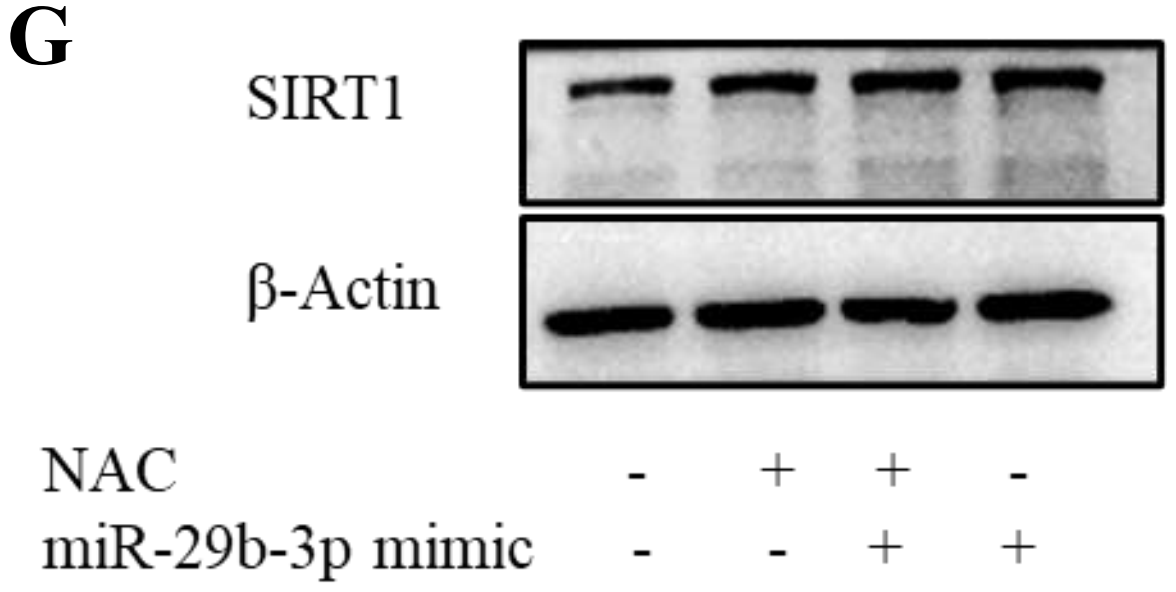

H

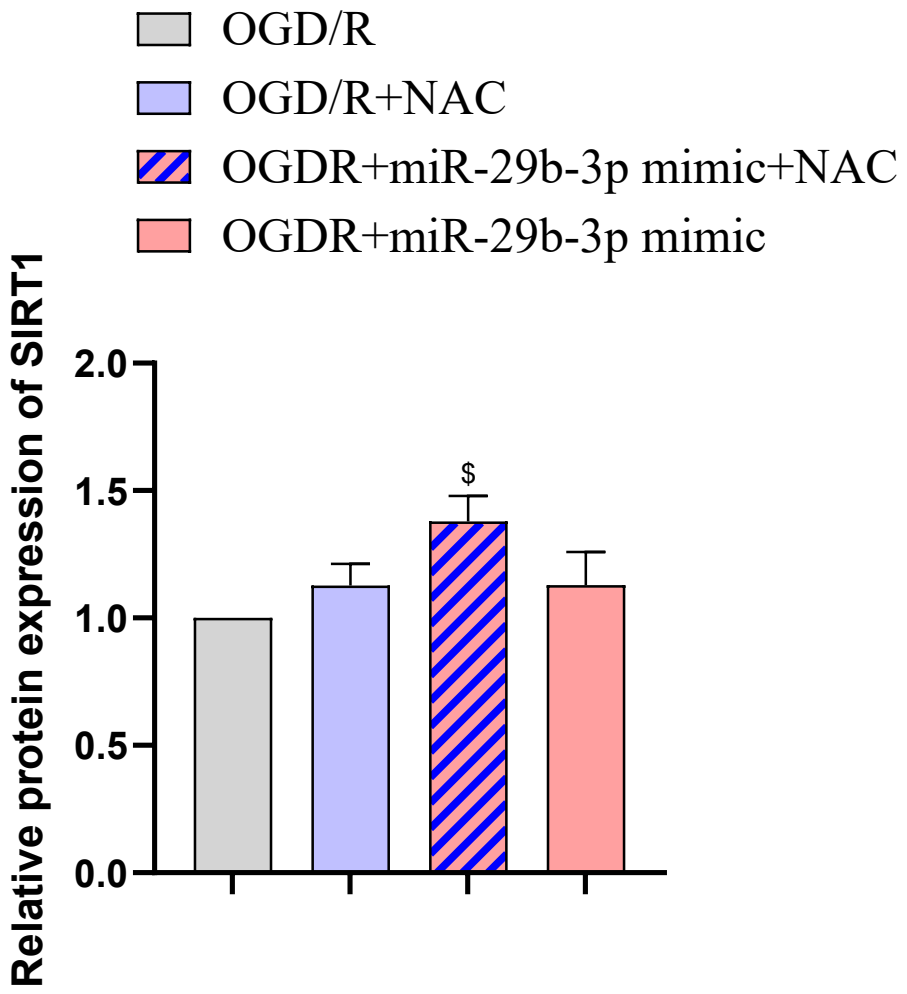

Figure 7G,7H. Determination of SIRT1 protein expression with NAC treatment by western blotting and calculated with reference to β-Actin. The values are expressed as the mean ± SD (n=3). $^{\$}$*P<0.05* vs. OGD/R+miR-29b-3p mimic.

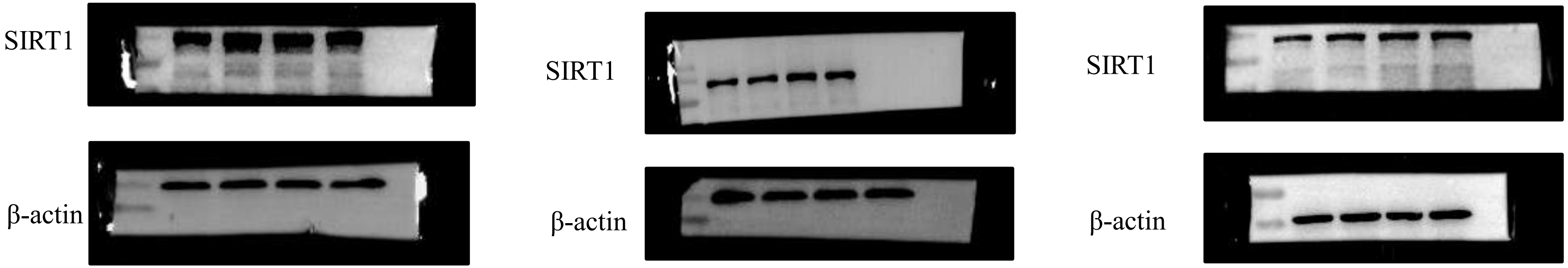

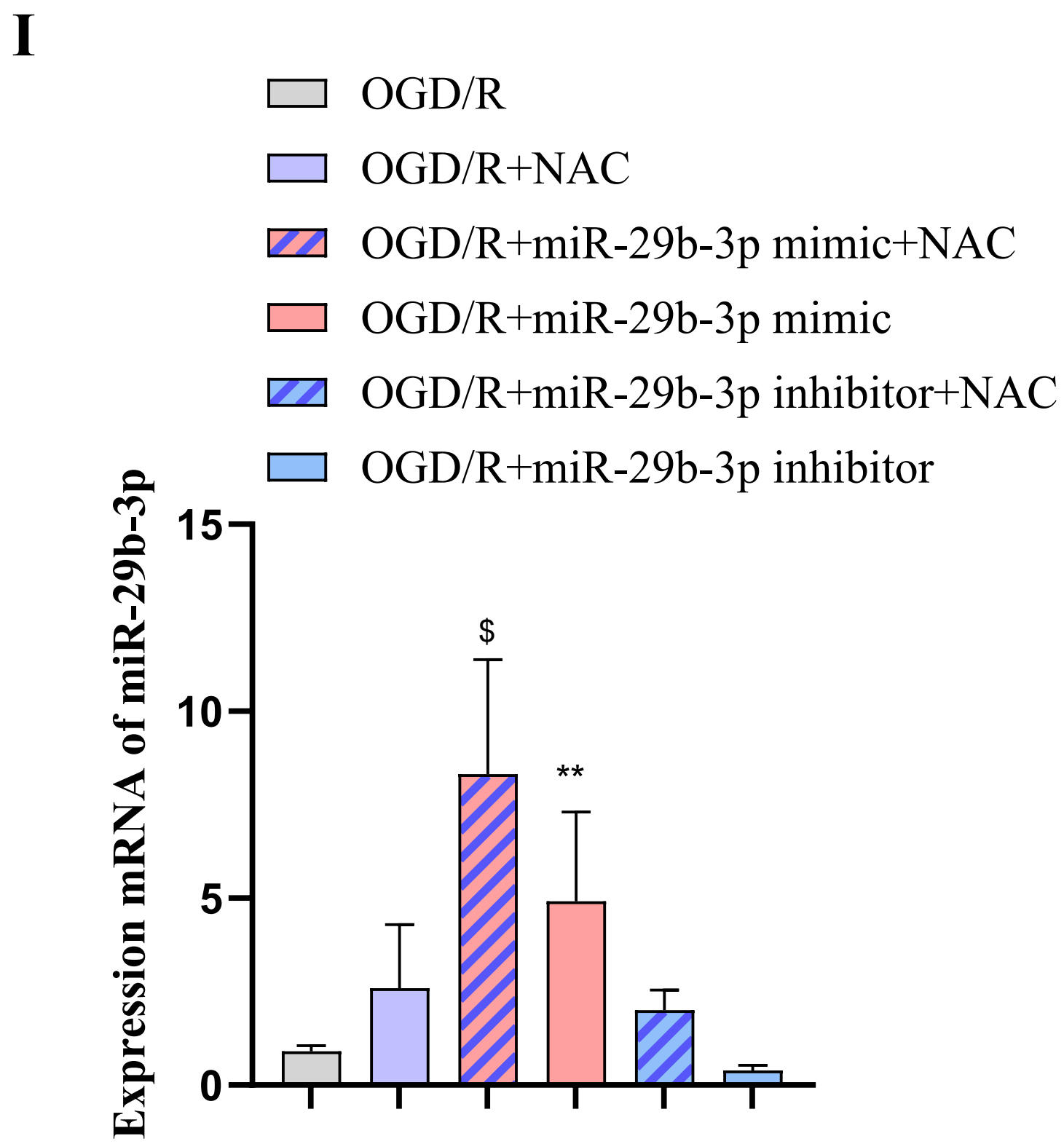

Figure 7I. NAC regulates the expression of miR-29b-3p. (**P<0.01 vs. OGD/R, $^{\$}$P<0.05 vs. OGD/R+miR-29b-3p mimic).
